# Comparison of Efficacy and Safety of Third-Line Treatments for Advanced Gastric Cancer: A Systematic Review With Bayesian Network Meta-Analysis

**DOI:** 10.3389/fonc.2021.734323

**Published:** 2021-10-22

**Authors:** Miao Huang, Jisheng Li, Xuejun Yu, Qian Xu, Xue Zhang, Xin Dai, Song Li, Lei Sheng, Kai Huang, Lian Liu

**Affiliations:** ^1^ Department of Medical Oncology, Qilu Hospital, Cheeloo College of Medicine, Shandong University, Jinan, China; ^2^ Department of Medical Oncology, Shandong Provincial Hospital of Traditional Chinese Medicine, Jinan, China; ^3^ Department of Thyroid Surgery, General Surgery, Qilu Hospital, Cheeloo College of Medicine, Shandong University, Jinan, China

**Keywords:** gastric cancer, third-line, chemotherapy, immune checkpoint inhibitors, anti-angiogenic therapy, network meta-analysis

## Abstract

**Background:**

Although various third-line treatments of advanced gastric cancer (AGC) significantly improved the overall survival, the optimal regimen has not been determined by now. This study aims to evaluate the efficacy and safety of multiple third-line treatments of AGC *via* integrated analysis and network meta-analysis (NMA) to provide valuable evidence for the optimal third-line systemic therapy for AGC.

**Methods:**

By searching the databases of PubMed, Embase and the Cochrane Central Register of Controlled Trials from Jan 01, 2005 to Dec 31, 2020, we included phase II/III randomized clinical trials (RCTs) of the third-line treatments for AGC to perform NMA. The main outcomes for NMA were median overall survival (mOS), median progression-free survival (mPFS), disease control rate (DCR) and adverse events (AEs). We also included phase IB/II non-RCTs and II/III RCTs of the third-line immune checkpoint inhibitors (ICIs) for integrated analysis for pooled mOS (POS), pooled mPFS (PPFS) and other outcomes.

**Results:**

Eight phase II/III RCTs and 2 ICIs-related phase IB/II non-RCTs were included for analysis, involving 9 treatment regimens and 3012 AGC patients. In terms of mOS, apatinib (hazard ratio [HR] 0.61, 95% credible interval [CrI] 0.48-0.78) and nivolumab (HR 0.62, 95% CrI 0.51-0.76) were the most effective treatments compared with placebo. Apatinib also significantly improved mPFS versus placebo (HR 0.38, 95% CrI 0.29-0.49). Nivolumab ranked first among all regimens for 1-year OS rate and achieved the best OS in patients with HER-2 positive tumor, patients with gastroesophageal junction (GEJ) cancer and patients without gastrectomy history. TAS-102 (OR 7.46, 95% CrI 4.61-12.51) was the most toxic treatment in terms of AEs of grade 3 and higher (≥3 AEs). Pembrolizumab was more likely to cause immune related adverse event. Finally, the POS, pooled 1-year OS rate, pooled ORR and PPFS of AGC patients treated with third-line ICIs were 5.1 months, 25%, 10% and 1.71 months respectively.

**Conclusions:**

Apatinib and nivolumab are the most effective treatments for the third-line treatment of AGC in contrast to the third-line chemotherapy. For AGC patients with HER-2 positive tumor, patients with GEJ cancer and patients without gastrectomy history, ICIs could be the optimal third-line treatment choice.

## Introduction

Gastric cancer (GC) is the fifth most common cancer type in the world and the fourth leading cause of cancer-related deaths, with 1 million newly diagnosed gastric cancer patients and an estimated 769,000 deaths worldwide in 2020 (1). Even after radical gastric resection, about 40-80% of operable GC of early clinical stage will still experience disease relapse (2). Moreover, about 50% of GC patients are diagnosed with advanced stage disease at the first medical visit with a quite dismal prognosis (3). Even with the recent progress in surgical and systemic treatments and the increasing great emphasis on multidisciplinary evaluation and treatment for gastric cancer, its global 5-year survival rate are still quite unsatisfying (23.7–26.2%) and advanced gastric cancer (AGC) only has a median overall survival (mOS) of 9–10 months (3, 4). For unresectable AGC, two meta-analysis studies confirmed that first-line and second-line chemotherapy significantly prolonged the overall survival (OS) of patients with AGC compared with best supportive care (BSC) (5, 6).

As for the third-line treatment of AGC, the phase III randomized clinical trial (RCT) by Kang JH et al. confirmed that irinotecan or docetaxel monotherapy significantly prolonged survival as third-line therapy when compared with BSC (7). A novel chemotherapy drug trifluridine/tipiracil (TAS-102) also significantly improved patient survival with a mOS of up to 5.7 months in third-line setting in AGC as shown in the TAGS study (8). The potential of anti-angiogenic therapy in the third-line treatment of AGC has been explored in two RCTs carried out in China (9, 10). Apatinib, a novel tyrosine kinase inhibitor (TKI) targeting VEGFR-2, was demonstrated with a statistically significant survival advantage in both OS and progression-free survival (PFS) compared with placebo and thus was approved as one of the standard third-line treatment selections in China (9, 10). However, in its global phase III ANGEL study (11), apatinib plus BSC failed to reveal significant OS benefit compared with placebo plus BSC in third-line or later-line treatment setting of AGC.

In the era of cancer immunotherapy, the third-line treatment algorithm for AGC has been constantly enriched and optimized along with the development and application of immune checkpoint inhibitors (ICIs). In the KEYNOTE-012 study, for the first-time anti-PD-1 antibody pembrolizumab was proved to be an effective third-line and later-line treatment of AGC with a satisfying mOS of 11.4 months in PD-L1 positive AGC patients (12). The results of KEYNOTE-059 study cohort 1 showed that pembrolizumab monotherapy as third-line and later-line treatment achieved a mOS of 5.6 months in PD-L1 ≥1% AGC population (13). Meanwhile, the phase III ATTRACTION-2 study carried out in Asia also demonstrated that OS of patients in the nivolumab monotherapy group was 5.26 months which was significantly better than that of the placebo group (14). As for anti-PD-L1 antibodies, the JAVELIN Gastric 300 study showed that avelumab monotherapy failed to improve OS or PFS when compared with physician’s choice of standard chemotherapy as third-line treatment, such as paclitaxel and irinotecan (15).

Recently, a systematic review and meta-analysis has confirmed that the third-line treatments for AGC or gastroesophageal junction (GEJ) cancer could significantly improve overall efficacy when compared with BSC (16, 17). Meanwhile, a lately reported network meta-analysis (NMA) of the third-line treatments in AGC demonstrated nivolumab in combination with ipilimumab to be the most effective therapeutic regimen with significant improvement of both OS and objective response rate (ORR) when compared with BSC. However, it’s quite noteworthy that the toxicity of this combination was the highest among all regimens (18). In addition, the supporting evidence for this nivolumab and ipilimumab combination in third-line setting came from a phase IB/II Checkmate-032 study instead of a solid phase III study (19). Moreover, in addition to AGC patients, patients with advanced esophageal cancer were also included in the Checkmate-032 study. Therefore, as a conclusion, the authors of above NMA recommended immunotherapy (nivolumab) or anti-angiogenic agents (regorafenib and apatinib) to be beneficial options in third-line and later-line treatment of AGC instead of the nivolumab and ipilimumab combination. Actually, the optimal strategy for the third-line treatments of AGC is still controversial at present and remains as an urgent problem to be solved in clinical practice (20). Therefore, we performed a systematic review and NMA based on most updated RCTs in order to comprehensively evaluate the effectiveness and safety of diverse third-line treatments in AGC and to provide valuable clinical reference and evidence for the third-line treatment of AGC.

## Materials And Methods

### Search Strategy and Eligibility Criteria

This NMA was performed according to the PRISMA extension statement (**Supplementary Table 1**) (21). By searching the databases of PubMed, Embase and the Cochrane Central Register of Controlled Trials from Jan 01, 2005 to Dec 31, 2020, we sought for published full text articles about the third-line treatments of AGC. For multiple reported data of an outcome from the same trial, only the latest data was kept. The detailed search strategy was shown in **Supplementary Table 2**.

We included phase II/III RCTs of the third-line treatments for AGC to perform NMA. And we also included phase IB/II non-RCTs and II/III RCTs of the third-line ICIs for integrated analysis of pooled median OS (POS), pooled median PFS (PPFS) and other outcomes. These trials met the following inclusion criteria: 1) Histologically confirmed AGC/GEJ cancer; 2) Single-arm studies that included third-line ICIs and two or more different-arm studies that included any third-line treatments; 3) The hazard ratio (HR) or odds ratio (OR) and its 95% credible interval (CrI) of OS, PFS, disease control rate (DCR) and adverse events (AEs) were available; 4) Published articles were reported in English.

Exclusion criteria: 1) Trials involving the results of radiotherapy, immune cells or cytokines, cancer vaccines, oncolytic viruses, and so on; 2) Trials only including results from special patient populations, such as patients with poor ECOG scores or elderly patients; 3) Research for which the final results have not been published or the published data was insufficient for analysis.

### Data Extraction and Risk of Bias Assessment

We extracted study ID, first author, publication year, journal of publication, sample size and outcomes of each treatment into a spreadsheet for further analysis. For AEs, we tended to use treatment-related adverse events (TRAEs) for analysis. When TRAEs were not reported, we used common AEs instead. The bias risk of included trials was assessed with the Cochrane Risk of Bias Tool including the following seven aspects: random sequence generation, allocation concealment, blinding of participants and personnel, blinding of outcome assessment, incomplete outcome data, selective outcome reporting and other sources of bias. Two investigators (MH and QX) independently conducted data extraction and assessed risk of bias of individual studies. Any disagreements were resolved through discussion and negotiation.

### Statistical Analysis

The primary outcome of this study was mOS. Secondary outcomes were mPFS, DCR and ≥ 3 AEs. In Stata (version 16.0), we performed integrated analysis on POS and PPFS of ICIs. NMA were performed in a Bayesian framework using a Markov Chain Monte Carlo simulation technique within the GEMTC package in the R-Statistics and the J.A.G.S. program (22). For each outcome, 150,000 sample iterations were generated with 100,000 burn-ins and a thinning interval of 10 (23). Fixed and random effect models were considered and compared using deviance information criteria (DIC). If the DIC difference between the random model and the fixed model was less than 5, the fixed model should be selected (24). Model convergence was assessed using a Brooks-Gelman-Rubin diagnostic plot and trace plot (25). Heterogeneity was assessed between studies using the I^2^ statistic. The estimated I^2^ values under 25%, between 25% and 50%, or over 50% indicated low, moderate, or high heterogeneity, respectively (26). All treatments were ranked according to the surface under the cumulative ranking curve (SUCRA). The higher SUCRA value meant that a treatment was more likely to be ranked on the top (27). Because the dose of apatinib was 850 mg once daily in its phase III study for third-line treatment in AGC, we only extracted the data of apatinib 850 mg once daily in its phase II study for NMA (9).

## Results

### Literature Search and Study Characteristics

**Figure 1:**
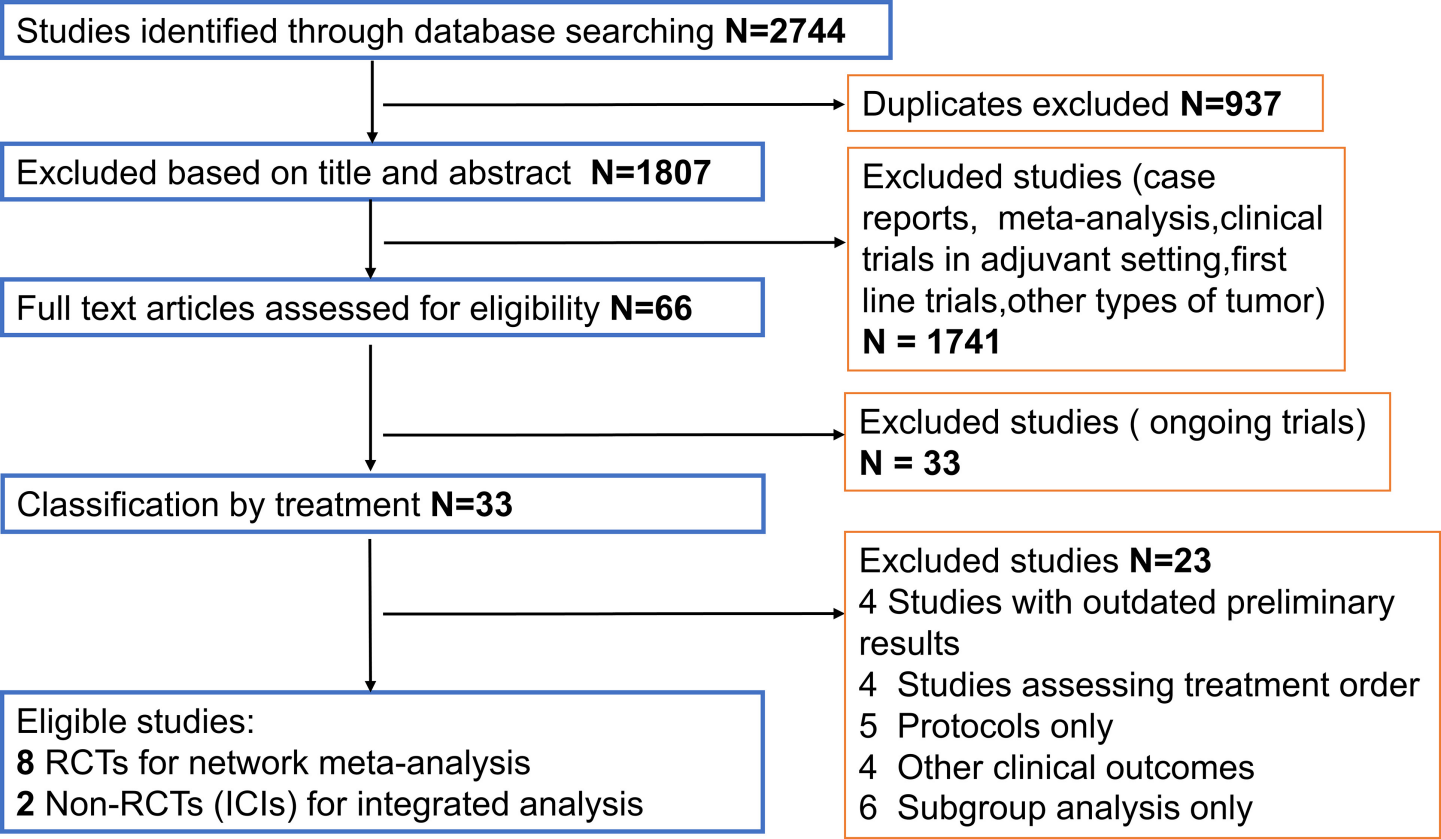
Study selection. ICIs, Immune checkpoint inhibitors.

**Figure 2:**
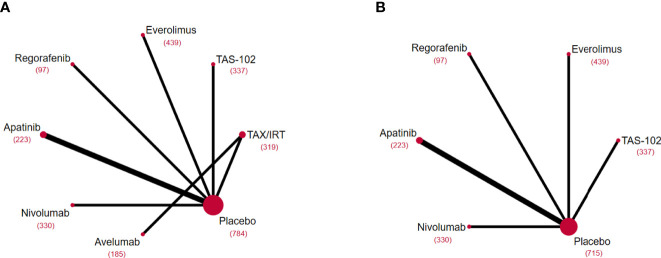
Network diagrams of comparisons on different outcomes of treatments included in the network meta-analysis of the third-line treatments for advanced GC/GEJ cancer. Comparisons on overall survival (OS) **(A)** and progression free survival (PFS) **(B)**. Each circular node represented a type of treatment. Each line represented a type of head-to-head comparison. The size of the nodes and the thickness of the lines were weighted according to the number of studies evaluating each treatment and direct comparison, respectively. The total number of patients receiving a treatment was shown in brackets. TAX, Docetaxel/Paclitaxel; IRT, Irinotecan; TAS-102, Trifluridine/Tipiracil; GC/GEJ cancer, Gastric cancer/Gastroesophageal junction cancer.

The flow chart depicting the study selection process was shown in **Figure 1**. We finally included 8 phase II/III RCTs (7–10, 14, 15, 28–30) and 2 ICIs-related phase IB/II non-RCTs (12, 13), involving 3012 patients and a total of 9 treatment regimens. The treatments included chemotherapy agents (taxol/irinotecan and trifluridine/tipiracil), targeted agent (everolimus), anti-angiogenic agents (apatinib and regorafenib), ICIs (nivolumab, pembrolizumab and avelumab). The network plot for direct and indirect comparison of all treatments were shown in **Figure 2**. The baseline characteristics of studies were shown in **Table 1**.

**Table 1:** Baseline characteristics of studies included in the systematic review with Bayesian network meta-analysis of third-line treatments for advanced gastric cancer.

Study(phase)	Country	Study design	Sample size;Median age	Male/Female	Intervention arm	Control arm	Reported outcomes	First-line treatment	Second-line treatment
**Kang JH et al.** **(III,** 7 **)**	Korea	RCT	133/69; 56/56	137/65	Single agent docetaxel 60 mg/m2 on D1 every 3 weeks or irinotecan 150 mg/m2 every 2 weeks	BSC	OS, PFS	Platinum and fluoropyrimidine	NA
**Shitara K et al.** **(III,** 8 **)**	17 countries worldwide	RCT	337/170; 64/63	369/138	Oral trifluridine/tipiracil 35 mg/m²/bid+BSC(on days 1–5 and days 8–12 of each 28-day treatment cycle)	Oral Placebo+BSC	OS, PFS, ORR, AE, DCR	Fluoropyrimidine, platinum, taxane, trastuzumab added for patients with HER-2+ tumors	Taxanes, irinotecan, or ramucirumab alone or in combination with paclitaxel
**Ohtsu A et al.** **(III,** 28 **)**	23 countries worldwide	RCT	439/217; 62/62	483/173	Oral everolimus 10 mg/d+BSC	Oral Placebo+BSC	OS, PFS, ORR, AE, DCR	Fluoropyrimidine, platinum and taxane	NA
**Pavlakis N et al.** **(II, ** 29 **)**	Australia, New Zealand,Canada,South Korea	RCT	97/50; 63/62	118/29	Oral regorafenib 160 mg daily on days 1–21 each 28-day cycle + BSC	Oral Placebo+BSC	PFS, OS, AE, ORR	NA	NA
**Li J et al.** **(II,** 9 **)**	China	RCT	47/24; 55/54	55/16	Oral apatinib 850 mg once daily (group B)	Oral placebo (group A)	PFS, OS, ORR, AE, DCR	Fluoropyrimidine, platinum and taxane	Taxanes and irinotecan
**Li J et al.** **(III,** 10 **)**	China	RCT	176/91; 58/58	201/66	Oral apatinib 850 mg once daily	Oral placebo	PFS, OS, ORR, AE, DCR	Fluoropyrimidine, platinum and taxane	Taxanes and irinotecan
**Kang YK et al. and Chen LT et al.** **(III,** 14, 30**)**	Japan, Korea, Taiwan	RCT	330/163; 62/61	348/145	Nivolumab 3 mg/kg every 2 weeks	Placebo	OS, PFS, AE, ORR, DCR	Platinum and pyrimidine analogues	Docetaxel, paclitaxel, or irinotecan monotherapy, ramucirumab alone or in combination with paclitaxel
**Bang YJ et al.** **(III,** 15 **)**	America,Asia, Australia, and Europe, USA	RCT	185/186; 59/61	267/104	Avelumab 10 mg/kg by intravenous infusion every 2 weeks	Paclitaxel 80 mg/m2 on days 1, 8, and 15 or irinotecan 150 mg/m2 on days 1 and 15	OS, PFS, ORR, DCR, AE	Platinum and fluoropyrimidine is standard, with trastuzumab added for patients with HER-2+ tumors	Taxanes, irinotecan, or ramucirumab alone or in combination with paclitaxel
**Fuchs CS et al.** **(II,** 13 **)**	52 sites in 16 countries	Non-RCT	259; 62	198/61	Pembrolizumab 200 mg on day 1 of each 3-week cycle	NA	ORR, OS, PFS, DCR	Platinum and fluoropyrimidine	Ramucirumab, alone or combined with a taxane or irinotecan
**Muro K et al.** **(1b,** 12 **)**	USA, Israel, Japan, South Korea, and Taiwan	Non-RCT	39 63	28/11	Intravenous pembrolizumab at 10 mg/kg once every 2 weeks for 24 months	NA	ORR, AE, OS, PFS, DCR	NA	Ramucirumab, alone or combined with a taxane or irinotecan

NA, not available; BSC, best supportive care; RCT, randomized clinical trial; Non-RCT, non-randomized clinical trial; OS, overall survival; PFS, progression-free survival; ORR, objective response rate; DCR, disease control rate; AE, adverse event; D1, day1; HER-2+,human epidermal growth factor receptor-2 positive.

### Overall Outcomes

**Figure 3:**
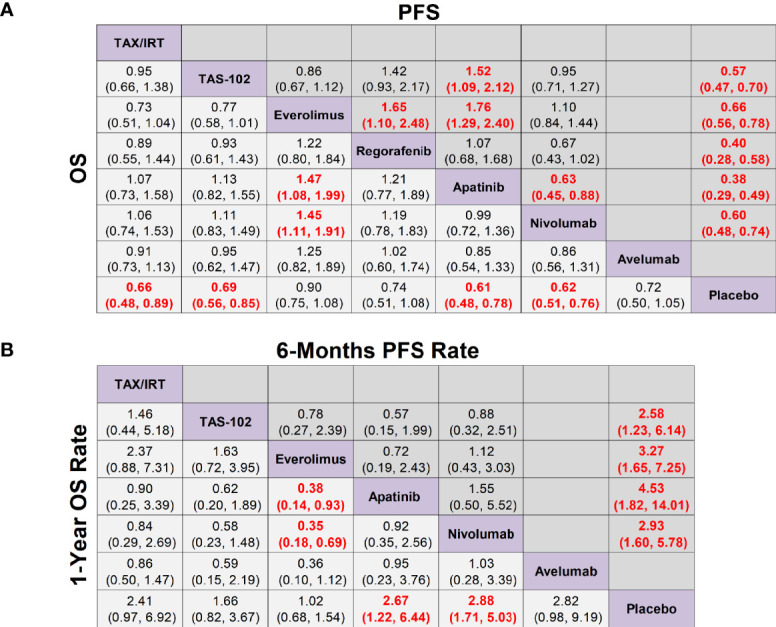
Network meta-analysis of the third-line treatments for advanced GC/GEJ cancer. **(A)** Pooled hazard ratio (HR) [95% credible intervals (CrI)] for overall survival (OS) and progression free survival (PFS). **(B)** Pooled odds ratio (OR) (95% CrI) for 6-months PFS rate and 1-year OS rate. Data in each cell are HR or OR (95% CrI) for the comparison of row-defining treatment versus column-defining treatment. HR less than 1 and OR more than 1 favored upper-row treatment. Significant results were highlighted in red and bold. TAX, Docetaxel/Paclitaxel; IRT, Irinotecan; TAS-102, Trifluridine/Tipiracil; GC/GEJ cancer, Gastric cancer/Gastroesophageal junction cancer.

In terms of OS, apatinib (HR 0.61, 95% CrI 0.48-0.78), nivolumab (HR 0.62, 95% CrI 0.51-0.76), taxol/irinotecan (HR 0.66, 95% CrI 0.48-0.89), TAS-102 (HR 0.69, 95% CrI 0.56-0.85) were all significantly better than placebo, but there was no significant difference among these four treatments (**Figure 3A**). According to the SUCRA results, the SUCRA values of apatinib (0.78) and nivolumab (0.77) were almost the same in OS and meant the highest probability of ranking first followed by taxol/irinotecan and then TAS-102 (**Supplementary Figure 1A**). Avelumab, everolimus and regorafenib exhibited no significant difference in OS compared with placebo (**Figure 3A**). In terms of PFS, apatinib (HR 0.38, 95% CrI 0.29-0.49), regorafenib (HR 0.40, 95% CrI 0.28-0.58), TAS-102 (HR 0.57, 95% CrI 0.47-0.70), nivolumab (HR 0.60, 95% CrI 0.48-0.74) and everolimus (HR 0.66, 95% CrI 0.56-0.78) showed significantly better efficacy over placebo and their rankings decreased sequentially. Moreover, apatinib (HR 0.63, 95% CrI 0.45-0.88) was associated with significantly longer PFS than nivolumab. The PFS of everolimus was significantly shorter than that of regorafenib (HR 1.65, 95% CrI 1.10-2.48) and apatinib (HR 1.76, 95% CrI 1.29-2.40) (**Figure 3A** and **Supplementary Figure 1A**).

As shown in **Figure 3B**, the 1-year OS rates of nivolumab (OR 2.88, 95% CrI 1.71-5.03) and apatinib (OR 2.67, 95% CrI 1.22-6.44) were significantly longer than placebo by 2.5 to 3 times, while the 1-year OS rates of chemotherapy agents taxol/irinotecan (OR 2.41, 95% CrI 0.97-6.92) and TAS-102 (OR 1.66, 95% CrI 0.82-3.67) had no statistical difference compared with placebo. In terms of 6-months PFS rate, apatinib (OR 4.53, 95% CrI 1.82-14.01), everolimus (OR 3.27, 95% CrI 1.65-7.25), nivolumab (OR 2.93, 95% CrI 1.60-5.78), TAS-102 (OR 2.58, 95% CrI 1.23-6.14) were significantly better than placebo, and the ranking declined sequentially.

In terms of ≥3 AEs, compared with placebo, TAS-102 was associated with the highest incidence rate of adverse events (OR 7.46, 95% CrI 4.61-12.51) followed by nivolumab (OR 3.07, 95% CrI 1.40-7.80). Although the incidence of ≥3 AEs of TAS-102 (OR 3.95, 95% CrI 1.67-9.40) was much more than that of regorafenib, regorafenib (OR, 1.89 95% CrI 0.93-3.83) actually did not significantly increase toxicity versus placebo. It’s worth noting that apatinib could cause a higher incidence of hand-foot syndrome (3.9%), but its gastrointestinal toxicity (0.5%) and hematological toxicity (1.9%) only accounted for a quite low proportion in NMA. For DCR, apatinib (OR 7.84, 95% CrI 4.12-16.50) was the best treatment followed by TAS-102 (OR 4.76, 95% CrI 2.86-8.12), everolimus (OR 2.72, 95% CrI 1.84-4.09), and nivolumab (OR 2.02, 95% CrI 1.27-3.25) (**Supplementary Figures 2**, **4A**).

**Figure 4:**
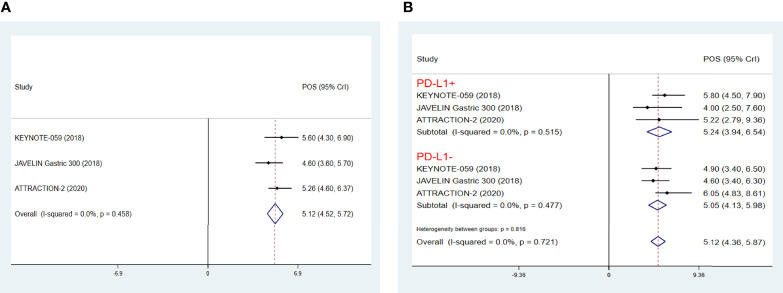
Pooled survival outcomes from integrated analysis of median overall survival (mOS) **(A)**, PDL1+/- mOS **(B)** of immune checkpoint inhibitors in patients with advanced GC/GEJ cancer. GC/GEJ cancer, Gastric cancer/Gastroesophageal junction cancer.

In view of the fact that some of the studies of ICIs as third-line treatments for AGC were phase IB/II studies without control groups and thus could not be included in NMA, we further performed an integrated analysis of the efficacy of ICIs, which has been the hot spot of clinical study in multiple cancer types including AGC. As a result, it was found that the POS of ICIs as third-line treatments for AGC was 5.12 months (95% CrI 4.52-5.72) **(** **Figure 4A** **)**, with a POS of PD-L1+ patients treated by ICIs of 5.24 months (95% CrI 3.94-6.54), a POS of PD-L1- patients of 5.05 months (95% CrI 4.13-5.98) (**Figure 4B**), a PPFS of 1.71months (95% CrI 1.26-2.16) (**Supplementary Figure 3A**), a pooled ORR of 10% (95% CrI 3%-17%) (**Supplementary Figure 3B**), a pooled 1-year OS rate of 25% (95% CrI 19%-31%) (**Supplementary Figure 3C**), and a pooled 1-year PFS rate of 8% (95% CrI 1%-15%) (**Supplementary Figure 3D**). In terms of TRAE, patients treated with pembrolizumab had the highest incidence of anemia, fatigue, hypothyroidism and arthralgia, while patients treated with nivolumab tended to have more incidence of pruritus. In addition, pembrolizumab was more likely to cause irAE. Meanwhile, nivolumab was more likely to cause interstitial lung disease (**Supplementary Figures 3E, F**).

### NMA of Histopathology or HER-2 Positive Subgroup

**Figure 5:**
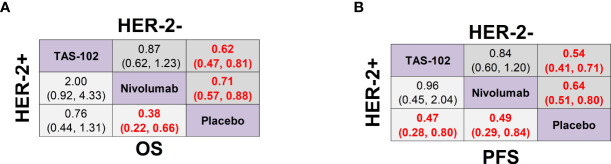
Network meta-analysis of the third-line treatments for GC/GEJ cancer based on HER-2 status. **(A)** Pooled HR (95% CrI) for OS of patients with human epidermal growth factor receptor-2 positive (HER-2+) and negative (HER-2-) tumors. **(B)** Pooled HR (95% CrI) for PFS of patients with HER-2+ and HER-2- tumors. GC/GEJ cancer, Gastric cancer/Gastroesophageal junction cancer.

In terms of Lauren classification, TAS-102 (HR 0.58, 95% CrI 0.39-0.87) and nivolumab (HR 0.62, 95% CrI 0.44-0.87) achieved significant OS advantages compared with placebo in the intestinal type of GC. However, the diffuse type of GC there was no significant difference in OS between all treatments and placebo (**Supplementary Figure 4B**). In the HER-2 positive subgroup analysis, nivolumab treatment significantly prolonged the OS of both HER-2+ (HR 0.38, 95% CrI 0.22-0.66) patients and HER-2- (HR 0.71, 95% CrI 0.57-0.88) patients compared with placebo, as well as the PFS of HER-2+ (HR 0.49, 95% CrI 0.29-0.84) patients and HER-2- (HR 0.64, 95% CrI 0.51-0.80) patients. TAS-102 also significantly prolonged the PFS of both HER-2+ (HR 0.47, 95% CrI 0.28-0.80) and HER-2- (HR 0.54, 95% CrI 0.41-0.71) patients. However, it only significantly prolonged the OS of HER-2- (HR 0.62, 95% CrI 0.47-0.81) patients but not the OS of HER-2+ (HR 0.76, 95% CrI 0.44-1.31) patients. In addition, there was no statistical difference in OS or PFS between TAS-102 and nivolumab in terms of different HER-2 status (**Figures 5A, B**).

### NMA of Previous Gastrectomy or Primary Sites Subgroup

As shown in **Supplementary Figure 4C**, for subgroup analyses with gastrectomy history, TAS-102 (HR 0.57, 95% CrI 0.41-0.79) and nivolumab (HR 0.61, 95% CrI 0.47-0.79) treatment achieved significant OS benefits compared with placebo. However, in the patient subgroup in absence of gastrectomy, only nivolumab (HR 0.71, 95% CrI 0.51-0.99) treatment could significantly prolong OS compared with placebo. For the primary sites of GC, nivolumab significantly improved the OS of both patients with gastric origination cancer (HR 0.69, 95% CrI 0.56-0.86) and patients with GEJ cancer (HR 0.42, 95% CrI 0.20-0.89) versus placebo (**Supplementary Figure 4D**).

### NMA of Age, Gender or ECOG Subgroup

In the age subgroup, avelumab significantly prolonged the OS of patients ≥ 65 years old (HR 0.54, 95% CrI 0.31-0.96) over placebo while TAS-102 (HR 0.67, 95% CrI 0.51-0.89) and nivolumab (HR 0.70, 95% CrI 0.54-0.91) significantly improved the survival of patients <65 years old (**Supplementary Figure 4E**). In males, nivolumab (HR 0.60, 95% CrI 0.48-0.75), avelumab (HR 0.60, 95% CrI 0.37-0.95), taxol/irinotecan (HR 0.60, 95% CrI 0.41-0.87) all yielded superior OS than placebo and their efficacy were generally equivalent. In females, all treatments failed to significantly improve OS when compared with placebo (**Supplementary Figure 4F**). In patients with ECOG PS=0, taxol/irinotecan (HR 0.59, 95% CrI 0.38-0.90), nivolumab (HR 0.62, 95% CrI 0.43-0.89) and TAS-102 (HR 0.67, 95% CrI 0.47-0.96) all exhibited significant OS benefits compared with placebo; in patient with ECOG PS=1, nivolumab (HR 0.67, 95% CrI 0.53-0.84) and TAS-102 (HR 0.69, 95% CrI 0.53-0.89) treatment achieved significantly better OS than placebo (**Supplementary Figure 4G**).

### NMA of Number of Previous Regimens, Metastasis Sites or Measurable Lesion Subgroup

In the subgroup of AGC patients who experienced previous two lines of treatment, TAS-102 (HR 0.68, 95% CrI 0.47-0.98) and apatinib (HR 0.70, 95% CrI 0.49-0.99) showed significant OS superiority to placebo. However, nivolumab failed to significantly improve the OS of patients with previous two lines (HR 0.75, 95% CrI 0.47-1.20) of treatment or patients with three lines (HR 0.78, 95% CrI 0.57-1.07) of treatment (**Supplementary Figure 4H**). It was reported that nivolumab had achieved a significant OS prolongation in fourth-line treatment (HR 0.48, 95% CrI 0.35-0.66) (14), which would contribute to the prolongation of the integral OS value. In subgroups with different numbers of metastasis sites, taxol/irinotecan (HR 0.55, 95% CrI 0.33-0.92), TAS-102 (HR 0.68, 95% CrI 0.49-0.95), and apatinib (HR 0.70, 95% CrI 0.51-0.97) significantly prolonged OS in patients with one metastasis site compared to placebo. In patient subgroup with two metastasis sites, nivolumab (HR 0.60, 95% CrI 0.48-0.76), taxol/irinotecan (HR 0.63, 95% CrI 0.42-0.94), and TAS-102 (HR 0.71, 95% CrI 0.54-0.94) significantly improved OS versus placebo (**Supplementary Figure 4I**). In patients with no measurable lesion (31), taxol/irinotecan (HR 0.36, 95% CrI 0.20-0.67) and TAS-102 (HR 0.21, 95% CrI 0.09-0.50) significantly improved OS versus placebo and TAS-102 (HR 0.25,95% CrI 0.09-0.66) exhibited significantly superior OS benefit over nivolumab. In patients with measurable lesion, both nivolumab (HR 0.61, 95% CrI 0.49-0.76) and TAS-102 (HR 0.74, 95% CrI 0.59-0.93) significantly improved OS versus placebo (**Supplementary Figure 4J**).

### Rank Probabilities

The Bayesian ranking probabilities of the survival benefits and corresponding SUCRA of comparable treatments were shown in **Supplementary Figures 1A–E**. For general OS, the SUCRA values of apatinib and nivolumab were almost the same which meant the highest probability of ranking first. At the same time, apatinib ranked best in terms of PFS, 6-months PFS rate and DCR. Nivolumab also ranked first in the 1-year OS rate and in terms of the OS of patient subgroups with HER-2 positive tumor, no gastrectomy history, primary site of GEJ, two metastasis sites, ECOG PS=1, and measurable lesion. Taxol/irinotecan ranked third in the OS of general population and ranked first in the OS of the subgroup of patients with one metastasis site. TAS-102 ranked fourth in the OS of general population and ranked first in the OS of patient subgroups with HER-2 negative tumor, previous two lines of treatment, gastrectomy history, age <65 years old, diffuse GC, primary site of stomach, and no measurable lesion. Meanwhile, it had the most severe treatment related toxicity and poorest tolerance.

### Risk of Bias Assessment, Model Convergence, Heterogeneity and Inconsistency Analysis

For most RCT studies, the risk of bias was generally low. Risk of bias assessment graph was presented in **Supplementary Figure 5**. As shown by the trace plot and the Brooks-Gelman-Rubin diagnostic plot, the convergence of our selected model was acceptable (**Supplementary Figure 6**). In the primary and secondary outcomes, the statistical heterogeneity of each study was low and moderate (I^2^<50%; range from 1% to 50%), and the fit of the consistency model in most comparisons was similar to or better than that of the inconsistency model (**Supplementary Figure 7**).

## Discussion

According to previous meta-analysis, the third-line treatments for AGC could significantly prolong OS compared with placebo (16, 17). However, the JAVELIN Gastric 300 study showed that anti-PD-L1 antibody avelumab failed to improve OS or PFS as third-line treatment when compared with physician’s choice of chemotherapy (15). With the quick development of treatment strategies for AGC in recent years, several standard chemotherapy agents, anti-angiogenic therapies, novel targeted therapies and ICIs have greatly enriched the choices for third-line treatments of AGC. Nevertheless, the relative efficacy and safety between these different regimens are waiting for further comparison and analysis. Although Sejung Park et al. performed a NMA on the third-line treatments of AGC (18), this study included a phase IB/II study involving patients with esophageal cancer in addition to gastric cancer (19). And the results of HR and 95% CrI were estimated based on the survival curves in the phase IB/II study. Therefore, the overall results and conclusion of this study might have limited credibility. Herein, we performed a systematic review and NMA to provide clinicians with more accurate evidence in order to choose the optimal third-line treatment for AGC.

The main findings of the current study are listed as follows: 1) In terms of general OS, apatinib and nivolumab were the most effective therapy among all choices. AGC patients treated with third-line apatinib had the best PFS with relatively lower gastrointestinal and hematological toxicity. Meanwhile, nivolumab ranked first among all regimens for 1-year OS rate and achieved the best OS in patients with HER-2 positive tumor, GEJ cancer and no gastrectomy history. Thus, apatinib and nivolumab could be the optimal choice for third-line treatment of AGC; 2) The POS of AGC patients treated with third-line ICIs could reach about 5 months, which was not correlated with PD-L1 expression level. The pooled 1-year OS rate of the third-line ICIs treatment was 25% with a pooled ORR of 10% and PPFS of 1.71 months. In addition, pembrolizumab was more likely to cause irAE; 3) Chemotherapy agents including taxol/irinotecan and TAS-102 had limited effect for the prolongation of OS. And TAS-102 was associated with the most sever treatment related toxicities in terms of ≥3 AEs.

In subgroup analysis of the present study, we found that the 1-year OS rate of nivolumab was 2.5 to 3 times higher than that of placebo, while third-line chemotherapy agents such as taxol/irinotecan and TAS-102 failed to significantly improve the 1-year OS rate of AGC patients. These results exactly reflected the long-term survival advantage of immunotherapy as third-line treatment for AGC (32). In the group of AGC patients with HER-2 positive tumor, TAS-102 as a third-line treatment failed to improve OS compared with placebo. In contrast, third-line ICIs treatment could significantly improve the OS of this group of AGC patients, suggesting that ICIs had therapeutic advantage in HER-2 positive gastric cancer. The detailed mechanism of HER-2 induced anti-tumor immunity improvement is still unclear at present. However, the study by Shiying Wu et al. demonstrated that HER-2 could recruit AKT1 to directly phosphorylate TBK1 and prevent the TBK1/STING association, thereby inhibited cGAS/STING signaling pathway and improved the anti-tumor immunity in the tumor microenvironment (33). Another study revealed that there was a correlation between HER-2 status and TMB level and HER-2 positive tumors had higher TMB and therefore better immunotherapy treatment efficacy (34). In addition, the activated HER-2 signaling pathway significantly increased the level of chemokines which were intensely involved in the recruitment of immune cells, resulting in a higher infiltration rate of activated T cells and monocytes and a higher expression level of PD-L1 in tumor microenvironment (35). A phase II clinical trial showed that anti-PD-1 antibody pembrolizumab combined with anti-HER-2 monoclonal antibody trastuzumab and chemotherapy achieved a quite high ORR of 87%, mPFS of 13 months, and mOS of 27.3 months as first-line treatment in patients with HER-2 positive AGC/GEJ cancer (36). It has been reported that female AGC patients are usually associated with early age of onset, mostly signet ring cell carcinoma and poorly differentiated adenocarcinoma pathological types, and quite poor reaction to anti-cancer treatments (37, 38). Similarly, the subgroup analysis of gender in the present study suggested that there was no significant OS benefit with any third-line treatments in female AGC patients. In addition, it’s worth noting that although avelumab did not show an OS advantage in the whole study population, it was the best treatment favoring OS in male AGC patients as well as in patients ≥65 years old.

In terms of the primary site of AGC, nivolumab could significantly improve the OS of both groups of patients with cancer originating in stomach and GEJ, though it was shown to be more effective in patients with GEJ cancer. Previous studies have shown that GC in western countries tended to originate mostly in the GEJ (39). And it was also known that the pathological types of GEJ included a proportion of squamous cell carcinoma, which were prone to have a better response to immunotherapy compared with adenocarcinoma. These facts might thus contribute to the better OS benefit of GEJ cancer from nivolumab as suggested by the current study (40, 41). In the subgroup analysis for gastrectomy, both TAS-102 and nivolumab achieved significantly better OS in patients who experienced gastrectomy. However, in patients without gastrectomy, only nivolumab could significantly improve OS compared with placebo. These results might suggest that multiple treatments including chemotherapy and immunotherapy were effective in AGC patients who progressed form early-staged GC after gastrectomy. Meanwhile, only third-line immunotherapy could bring survival benefits to patients with an initial diagnosis of AGC and thus no opportunity of receiving radical gastric resection. Therefore, early detection, early diagnosis, and early treatment not even greatly improved the cure rate of GC but also enriched the choices of efficient subsequent treatment after recurrence, which would further prolong the survival of patients (42). In addition to TAS-102 and ICI monotherapy, the combination of ICI and anti-angiogenic agent in AGC has recently shown good prospect in multiple small-sized clinical studies (43–48). Small molecular VEGFR TKI lenvatinib in combination with anti-PD-1 antibody pembrolizumab achieved a satisfying ORR of 69% and mPFS of 7.1 months in the first-line or second-line treatment of GC as revealed in the phase 2 EPOC1706 study (43). However, in third-line setting, the ORR of lenvatinib and pembrolizumab combination was only 10% and the mOS was only 5.9 months in the GC cohort of LEAP-005 study (48). In addition, the NivoRam study demonstrated that VEGFR-2 antibody ramucirumab in combination with nivolumab obtained a mOS of 9 months as a second-line treatment for GC (44). Moreover, the ORR reached 44% and mPFS reached 5.6 months in the REGONIVO study, in which another small molecular VEGFR TKI regorafenib was combined with nivolumab as third-line or later-line treatment for GC (47). Therefore, although there are still some uncertainties with this novel combination, ICI combined with anti-angiogenic agent is quite likely to be a promising treatment strategy worthy of further confirmation in future studies.

In accordance with the results of previous systematic reviews and meta-analysis of the third-line treatments of AGC (16, 17, 20), in the present study we also found that the third-line treatments of AGC was generally better for patients’ survival than placebo. However, previous meta-analysis study only compared the third-line treatments with BSC, while NMA could analyze the difference of efficacy between various third-line treatments for AGC *via* direct and indirect comparisons. Furthermore, NMA based on Bayesian approaches was able to rank various treatment regimens according to the SUCRA (49). Therefore, the present NMA-based study could provide more precise evidence based on direct comparison and better decision-making algorithm for the third-line treatments of AGC. Similar to the NMA study of the third-line treatments in AGC by Sejung Park et al. (18), our results also demonstrated apatinib and nivolumab to be the best choices for third-line treatment of AGC in terms of overall efficacy. However, in the study by Sejung Park et al., although nivolumab combined with ipilimumab was the most effective treatment for OS improvement, its toxicity was the most severe among all regimens. As a conclusion, nivolumab, apatinib and regorafenib were recommended as the best options in their study (18). Unlike above study, results of the present study suggested that third-line apatinib and nivolumab significantly improved OS in AGC with less adverse events accounting only for a relatively low proportion of the entire NMA. Therefore, the evidence supporting apatinib and nivolumab as the optimal third-line treatment of AGC was more sufficient and valid in our study. In addition to the clinical studies included by Sejung Park et al., we included two extra studies for analysis (7, 9). Moreover, evidence supporting the role of nivolumab and ipilimumab combination in the NMA by Sejung Park et al. came from the phase IB/II Checkmate-032 study (19), in which esophageal cancer patients were also included and the exact values of HR and 95% CI were not reported. Due to above consideration, we believe that the results of Checkmate-032 study might affect the reliability of the NMA conclusions for third-line treatment of AGC and thus was not suitable to be included in our study.

The present study has the following limitations. First, because there was only one RCT evaluating each third-line therapeutic agent or combination in AGC, it might reduce the statistical power of the present study because of heterogeneity. However, no statistical heterogeneity was detected in the present study. Second, we did not perform nodal analysis based on Bayesian method or direct meta-analysis based on frequency method because closed loops could not be established in our NMA. Thus, we were unable to assess the inconsistency of the analysis arising from heterogeneity (50). Third, because most of the included RCTs enrolled mixed populations, we could not perform subgroup analysis based on Asians and non-Asians populations, which might be another potential source of heterogeneity. In addition, the diverse characteristics and criteria for enrollment in different studies might also lead to biased results. Fourth, because some of the 1-year OS rate and 6-months PFS rate data were extracted or calculated from the figures, the accuracy and reliability might be limited. Finally, in terms of the definition of PD-L1 positive, PD-L1 antibody types and TPS/CPS scores used were inconsistent among studies. In detail, tumors were considered PD-L1 positive if the combined positive score was 1 or greater with Merck 22C3 antibody in KEYNOTE-059 study (13), while in ATTRACTION-2 and JAVELIN Gastric 300 studies PD-L1 positive was defined as staining in 1% or more of tumor cells with Dako 28-8 antibody and Dako 73-10 antibody respectively (14, 15). Thus the validity of the pooled overall survival analysis result based on PD-L1 status was undermined due to these inconsistencies.

## Conclusions

In summary, apatinib and nivolumab seemed to be the optimal third-line AGC treatment methods at present base on the efficacy, safety and subgroup analysis results in the present study. Meanwhile, the efficacy of third-line chemotherapy agents was relatively limited in AGC. For AGC patients with HER-2 positive tumor, patients without gastrectomy history, and patients with primary GEJ cancer, ICIs were the best third-line treatment choice and could bring a mOS of 5 months for patients with AGC. Results of our study could help clinicians to choose the most appropriate third-line treatments option for AGC patients with different clinical characteristics for best survival with acceptable safety profile. Due to the limited number and quality of included clinical studies, these results need to be further confirmed by future RCTs with large sample sizes.

## Data Availability Statement

The original contributions presented in the study are included in the article/**Supplementary Material**. Further inquiries can be directed to the corresponding author.

## Author Contributions

LL is the corresponding author. LL contributed to the study concept and design. LL and MH took part in the initial literature search and assessed the eligibility of feasible studies. MH and JL interpreted the findings and wrote the first draft of the manuscript. MH, JL, XY, QX, XZ, XD, SL, LS, and KH prepared the figures and tables. LL and JL revised and edited the manuscript. All authors approved the final version of the manuscript. LL is the guarantor of this study and accepts full responsibility for the work, has access to the data, and controls the decision to publish. The corresponding authors attest that all listed authors meet authorship criteria and that no other person meeting the criteria has been omitted. All authors contributed to the article and approved the submitted version.

## Funding

This work was supported by the National Natural Science Foundation of China (81172487, 82173305 to LL and 81500092 to SL), Natural Science Foundation of Shandong Province (ZR2017MH005 to LL), and Foundation of Shandong University Clinical Research Center (2020SDUCRCC011 to SL).

## Conflict of Interest

The authors declare that the research was conducted in the absence of any commercial or financial relationships that could be construed as a potential conflict of interest.

## Publisher’s Note

All claims expressed in this article are solely those of the authors and do not necessarily represent those of their affiliated organizations, or those of the publisher, the editors and the reviewers. Any product that may be evaluated in this article, or claim that may be made by its manufacturer, is not guaranteed or endorsed by the publisher.
