# Supplementary material for: Comparison of Efficacy and Safety of Third-Line Treatments for Advanced Gastric Cancer: A Systematic Review With Bayesian Network Meta-Analysis
Source: Front Oncol. 2021 Oct 22;11:734323. doi: 10.3389/fonc.2021.734323 (PMC8570109; doi:10.3389/fonc.2021.734323)
Supplement: Supplementary file 1 [file DataSheet_1.pdf]

Supplementary Material

Supplementary Tables

Supplementary Table 1. Checklist of the PRISMA extension for network meta-analysis.

Section/topic	*#*	*Checklist item*	*Reported on page #*
**TITLE**			
Title	1	Identify the report as a systematic review, meta-analysis, or both.	1
**ABSTRACT**			
Structured summary	2	Provide a structured summary including, as applicable: background; objectives; data sources; study eligibility criteria, participants, and interventions; study appraisal and synthesis methods; results; limitations; conclusions and implications of key findings; systematic review registration number.	1,2
**INTRODUCTION**			
Rationale	3	Describe the rationale for the review in the context of what is already known. Including mention of why a network meta-analysis has been conducted.	2
Objectives	4	Provide an explicit statement of questions being addressed with reference to participants, interventions, comparisons, outcomes, and study design (PICOS).	3
**METHODS**			
Protocol and registration	5	Indicate if a review protocol exists, if and where it can be accessed (e.g., Web address), and, if available, provide registration information including registration number.	3
Eligibility criteria	6	Specify study characteristics (e.g., PICOS, length of follow-up) and report characteristics (e.g., years considered, language, publication status) used as criteria for eligibility, giving rationale.	3
Information sources	7	Describe all information sources (e.g., databases with dates of coverage, contact with study	3

Section/topic	*#*	*Checklist item*	*Reported on page #*
		authors to identify additional studies) in the search and date last searched.	
Search	8	Present full electronic search strategy for at least one database, including any limits used, such that it could be repeated.	3
Study selection	9	State the process for selecting studies (i.e., screening, eligibility, included in systematic review, and, if applicable, included in the meta-analysis).	3,4
Data collection process	10	Describe method of data extraction from reports (e.g., piloted forms, independently, in duplicate) and any processes for obtaining and confirming data from investigators.	4
Data items	11	List and define all variables for which data were sought (e.g., PICOS, funding sources) and any assumptions and simplifications made.	4
Risk of bias in individual studies	12	Describe methods used for assessing risk of bias of individual studies (including specification of whether this was done at the study or outcome level), and how this information is to be used in any data synthesis.	4
Summary measures	13	State the principal summary measures (e.g., risk ratio, difference in means). Also describe the use of additional summary measures assessed, such as treatment rankings and surface under the cumulative ranking curve (SUCRA) values, as well as modified approaches used to present summary findings from meta-analyses.	4
Synthesis of results	14	Describe the methods of handling data and combining results of studies, if done, including Assessment of model fit (e.g., I2) for each meta-analysis.	4
Assessment of Inconsistency	S2	Describe the statistical methods used to evaluate the agreement of direct and indirect evidence in the treatment network(s) studied. Describe efforts taken to address its presence when found.	4
Risk of bias across studies	15	Specify any assessment of risk of bias that may affect the cumulative evidence (e.g., publication bias, selective reporting within studies).	4
Additional analyses	16	Describe methods of additional analyses (e.g., sensitivity or subgroup analyses, meta-regression), if done, indicating which were pre-specified.	4
RESULTS			

Section/topic	*#*	*Checklist item*	*Reported on page #*
Study selection	17	Give numbers of studies screened, assessed for eligibility, and included in the review, with reasons for exclusions at each stage, ideally with a flow diagram.	4
Presentation of network structure	S3	Provide a network graph of the included studies to enable visualization of the geometry of the treatment network	4 (Figure 2)
Summary of network geometry	S4	Provide a brief overview of characteristics of the treatment network. This may include commentary on the abundance of trials and randomized patients for the differentinterventions and pairwise comparisons in the network, gaps of evidence in the treatment network, and potential biases reflected by the network structure.	4
Study characteristics	18	For each study, present characteristics for which data were extracted (e.g., study size, PICOS, follow-up period) and provide the citations.	4
Risk of bias within studies	19	Present data on risk of bias of each study and, if available, any outcome-level assessment .	7
Results of individual studies	20	For all outcomes considered (benefits or harms), present, for each study: (a) simple summary data for each intervention group and (b) effect estimates and confidence intervals, ideally with a forest plot.	5, 6, 7
Synthesis of results	21	Present results of each meta-analysis done, including confidence/credible intervals. In larger networks, authors may focus on comparisons versus a particular comparator (e.g. placebo or standard care), with full findings presented in an appendix. League tables and forest plots may be considered to summarize pairwise comparisons. If additional summary measures were explored (such as treatment rankings), these should also be presented.	5, 6, 7
Exploration for inconsistency	S5	Describe results from investigations of inconsistency. This may include such information as measures of model fit to compare consistency and inconsistency models, P values from statistical tests, or summary of inconsistency estimates from different parts of the treatment network.	7
Risk of bias across studies	22	Present results of any assessment of risk of bias across studies.	7
Additional analysis	23	Give results of additional analyses, if done (e.g., sensitivity or subgroup analyses, meta-	5, 6, 7

Section/topic	*#*	*Checklist item*	*Reported on page #*
		regression).	
DISCUSSION			
Summary of evidence	24	Summarize the main findings including the strength of evidence for each main outcome; consider their relevance to key groups (e.g., health care providers, users, and policy makers).	7, 8, 9
Limitations	25	Discuss limitations at study and outcome level (e.g., risk of bias), and at review level (e.g., incomplete retrieval of identified research, reporting bias).	9
Conclusions	26	Provide a general interpretation of the results in the context of other evidence, and implications for future research.	10
FUNDING			
Funding	27	Describe sources of funding for the systematic review and other support (e.g., supply of data); role of funders for the systematic review.	10

Supplementary Table 2. Literature search criteria.

A. Search strategy on Pubmed.

((((((((((((((("Stomach Neoplasms"[Mesh]) OR ("Gastric Neoplasms"[Title/Abstract])) OR ("Stomach Cancers"[Title/Abstract])) OR ("Gastric Cancer"[Title/Abstract])) OR ("gastroesophageal junction cancer"[Title/Abstract])) OR ("Cancer of the Stomach"[Title/Abstract])) OR ("adenocarcinoma of the stomach"[Title/Abstract])) OR ("adenocarcinoma of the gastroesophageal junction"[Title/Abstract])) OR (gastic cancer[Title/Abstract])) AND ((((((((((((((((((((((chemotherapy[Title/Abstract]) OR ("S-1"[Title/Abstract])) OR (TAS 102[Title/Abstract])) OR ((((((((((((((((((((((((((((((((((((((pembrolizumab[Title/Abstract]) OR (lambrolizumab[Title/Abstract])) OR (Nivolumab[Title/Abstract])) OR (atezolizumab[Title/Abstract])) OR ("Immunoglobulin G1"[Title/Abstract])) OR (durvalumab[Title/Abstract])) OR (avelumab[Title/Abstract])) OR (sintilimab[Title/Abstract])) OR (camrelizumab[Title/Abstract])) OR (toripalimab[Title/Abstract])) OR (Tislelizumab[Title/Abstract])) OR (Pidilizumab[Title/Abstract])) OR (Ipilimumab[Title/Abstract])) OR (Tremelimumab[Title/Abstract])) OR ("Immunotherapy"[Mesh])) OR ("immune checkpoint"[Title/Abstract])) OR (immunotherapy*[Title/Abstract])) OR (ICB[Title/Abstract])) OR ("Programmed Death-Ligand 1"[Title/Abstract])) OR ("Programmed Death 1"[Title/Abstract])) OR ("Programmed Cell Death 1 Receptor"[Mesh])) OR ("programmed cell death 1 ligand 1 protein"[Title/Abstract])) OR (antiPDL1[Title/Abstract])) OR (antiPD1[Title/Abstract])) OR ("PD-1"[Title/Abstract])) OR ("PD 1"[Title/Abstract])) OR (PD1[Title/Abstract])) OR ("PD-L1"[Title/Abstract])) OR ("PD L1"[Title/Abstract])) OR (PDL1[Title/Abstract])) OR ("CTLA-4 Antigen"[Mesh])) OR ("Cytotoxic T Lymphocyte Associated Antigen 4"[Title/Abstract])) OR ("LAG-3"[Title/Abstract])) OR ("TIM-3"[Title/Abstract])) OR (TIGIT[Title/Abstract])) OR (VISTA[Title/Abstract])) OR ("V-Domain Immunoglobulin-Containing Suppressor of T Cell Activation"[Title/Abstract]))) OR (((((((((VEGFR[Title/Abstract]) OR (Antiangiogenesis[Title/Abstract])) OR (apatinib[Title/Abstract])) OR (ramucirumab[Title/Abstract])) OR (Bevacizumab[Title/Abstract])) OR ("Receptor, ErbB-2"[Title/Abstract])) OR (Trastuzumab[Title/Abstract])) OR (pertuzumab[Title/Abstract])) OR (Endostar[Title/Abstract])) OR (Herceptin[Title/Abstract])) OR (taxane[Title/Abstract])) OR (irinotecan[Title/Abstract])) OR (Regorafenib[Title/Abstract]))) AND ((((((((((((((((((((3L[Title/Abstract]) OR ("3 Line"[Title/Abstract])) OR ("3 line"[Title/Abstract])) OR ("third line"[Title/Abstract])) OR ("previously treated"[Title/Abstract])) OR ("3-L"[Title/Abstract])) OR (3l[Title/Abstract])) OR ("3-Line"[Title/Abstract])) OR ("3-line"[Title/Abstract])) OR ("Third Line"[Title/Abstract])) OR ("Previously Treated"[Title/Abstract])) OR (treated[Title/Abstract])) OR (Treated[Title/Abstract])) OR ("third-line"[Title/Abstract])) OR ("Third-Line"[Title/Abstract])) OR ("later treated"[Title/Abstract])) OR ("Later Treated"[Title/Abstract])) OR (pretreated[Title/Abstract])) OR (placebo[Title/Abstract])) Filters: Clinical Trial, from 2005/1/1 - 2020/12/31

B. Search strategy on Embase.

#1 'stomach tumor'/exp OR 'stomach tumor':ti OR 'neoplasm, stomach':ti OR 'gastric neoplasms':ti OR 'stomach cancers':ti OR 'gastric cancer':ti OR 'gastroesophageal junction cancer':ti OR 'adenocarcinoma of the gastroesophageal junction':ti OR 'adenocarcinoma of the stomach':ti
#2 'chemotherapy':ti,ab OR 'tas 102':ti,ab OR 's-1':ti,ab OR 'pembrolizumab':ti OR 'lambrolizumab':ti OR 'nivolumab':ti OR 'atezolizumab':ti OR 'durvalumab':ti OR 'avelumab':ti OR 'sintilimab':ti OR 'camrelizumab':ti OR 'toripalimab':ti OR 'tislelizumab':ti OR 'pidilizumab':ti OR 'ipilimumab':ti OR 'tremelimumab':ti OR 'immunotherapy'/exp OR 'immune checkpoint':ti,ab OR 'immunotherapy*':ti,ab OR 'icb':ti,ab OR 'programmed death-ligand 1':ti,ab OR 'programmed death 1':ti,ab OR 'programmed death 1 receptor'/exp OR 'programmed cell death 1 ligand 1 protein':ti,ab OR 'antipdl1':ti,ab OR 'antipd1':ti,ab OR 'pd-1':ti,ab OR 'pd 1':ti,ab OR pd1:ti,ab OR 'pd-l1':ti,ab OR 'pd l1':ti,ab OR 'pdl1':ti,ab OR 'ctla-4 antigen':ti,ab OR 'cytotoxic t lymphocyte antigen 4'/exp OR 'lag-3':ti,ab OR 'tim-3':ti,ab OR 'tigit':ti,ab OR 'vista':ti,ab OR 'v-domain immunoglobulin-containing suppressor of t cell activation':ti,ab OR 'vegfr':ti,ab OR 'antiangiogenesis':ti,ab OR 'apatinib':ti,ab OR 'ramucirumab':ti,ab OR 'bevacizumab':ti,ab OR 'receptor, erbb-2':ti,ab OR 'trastuzumab':ti,ab OR pertuzumab:ti,ab OR endostar:ti,ab OR herceptin:ti,ab OR regorafenib:ti,ab
#3 3l:ti,ab OR '3 line':ti,ab OR 'third line':ti,ab OR 'previously treated':ti,ab OR '3-l':ti,ab OR '3-line':ti,ab OR 'treated':ti,ab OR 'third-line':ti,ab OR 'later treated':ti,ab OR pretreated:ti,ab OR placebo:ti,ab
#4 'clinical trial'
#1 AND #2 AND #3 AND #4 AND [english]/lim AND [1-1-2005]/sd NOT [1-1-2021]/sd AND [2005-2020]/py

C. Search strategy on Cochrane Central Register of Controlled Trials

#1 MeSH descriptor: [Stomach Neoplasms] explode all trees
#2 ("Neoplasm, Stomach"):ti,ab,kw OR ("Gastric Neoplasms"):ti,ab,kw OR ("Neoplasms, Gastric"):ti,ab,kw OR ("Stomach Cancers"):ti,ab,kw OR ("Gastric Cancer"):ti,ab,kw OR ("Neoplasm, Stomach"):ti,ab,kw OR ("Gastric Neoplasms"):ti,ab,kw OR ("Stomach Cancers"):ti,ab,kw OR ("Gastric Cancer"):ti,ab,kw
#3 ("Neoplasm, Stomach"):ti,ab,kw OR ("Cancer, Stomach"):ti,ab,kw OR ("gastroesophageal junction cancer"):ti,ab,kw OR ("Cancer of the Stomach"):ti,ab,kw OR ("Gastric Cancer, Familial Diffuse"):ti,ab,kw OR ("adenocarcinoma of the gastroesophageal junction"):ti,ab,kw OR ("adenocarcinoma of the stomach"):ti,ab,kw OR ("gastroesophageal junction cancer"):ti,ab,kw OR ("Cancer of the Stomach"):ti,ab,kw OR ("adenocarcinoma of the gastroesophageal junction"):ti,ab,kw OR ("adenocarcinoma of the stomach"):ti,ab,kw
#4 #1 OR #2 OR #3
#5 (chemotherapy):ti,ab,kw OR ("TAS 102"):ti,ab,kw OR ("S-1"):ti,ab,kw
#6 (pembrolizumab):ti,ab,kw OR (lambrolizumab):ti,ab,kw OR (Nivolumab):ti,ab,kw OR (atezolizumab):ti,ab,kw OR (Immunoglobulin G1):ti,ab,kw OR (durvalumab):ti,ab,kw OR (avelumab):ti,ab,kw OR (sintilimab):ti,ab,kw OR (camrelizumab):ti,ab,kw OR (toripalimab):ti,ab,kw OR (Tislelizumab):ti,ab,kw OR (Pidilizumab):ti,ab,kw OR (Ipilimumab):ti,ab,kw OR (Tremelimumab):ti,ab,kw
#7 MeSH descriptor: [Immunotherapy] explode all trees
#8 ("immune checkpoint"):ti,ab,kw OR (immunotherapy*):ti,ab,kw OR (ICB):ti,ab,kw OR ("Programmed Death-Ligand 1"):ti,ab,kw OR ("Programmed Death 1"):ti,ab,kw
#9 MeSH descriptor: [Programmed Cell Death 1 Receptor] explode all trees
#10 ("programmed cell death 1 ligand 1 protein"):ti,ab,kw OR (antiPDL1):ti,ab,kw OR (antiPD1):ti,ab,kw OR (PD-1):ti,ab,kw OR ("PD 1"):ti,ab,kw OR (PD1):ti,ab,kw OR (PD-L1):ti,ab,kw OR ("PD L1"):ti,ab,kw OR (PDL1):ti,ab,kw
#11 MeSH descriptor: [CTLA-4 Antigen] explode all trees
#12 ("Cytotoxic T Lymphocyte Associated Antigen 4"):ti,ab,kw OR (LAG-3):ti,ab,kw OR (TIM-3):ti,ab,kw OR (TIGIT):ti,ab,kw OR (VISTA):ti,ab,kw OR ("V-Domain Immunoglobulin-Containing Suppressor of T Cell Activation"):ti,ab,kw OR taxane:ti,ab,kw OR irinotecan:ti,ab,kw OR Regorafenib:ti,ab,kw
#13 (VEGFR):ti,ab,kw OR (Antiangiogenesis):ti,ab,kw OR (apatinib):ti,ab,kw OR (ramucirumab):ti,ab,kw OR (Bevacizumab):ti,ab,kw #14 ("Receptor, ErbB-2"):ti,ab,kw OR (Trastuzumab):ti,ab,kw OR (pertuzumab):ti,ab,kw OR (Endostar):ti,ab,kw OR (Herceptin):ti,ab,kw #15 #5 OR #6 OR #7 OR #8 OR #9 OR #10 OR #11 OR #12 OR #13 OR #14
#16 (3L):ti,ab,kw OR ("3 Line"):ti,ab,kw OR ("3 line"):ti,ab,kw OR ("third line"):ti,ab,kw OR ("previously treated"):ti,ab,kw #17 ("3-L"):ti,ab,kw OR (3l):ti,ab,kw OR ("3-Line"):ti,ab,kw OR ("3-line"):ti,ab,kw OR ("Third Line"):ti,ab,kw
#18 ("Previously Treated"):ti,ab,kw OR (treated):ti,ab,kw OR (Treated):ti,ab,kw OR ("third-line"):ti,ab,kw OR ("Third-Line"):ti,ab,kw #19 ("later treated"):ti,ab,kw OR ("Later Treated"):ti,ab,kw OR (pretreated):ti,ab,kw OR (placebo):ti,ab,kw
#20 #16 OR #17 OR #18 OR #19
#21 #4 AND #15 AND #20 with Cochrane Library publication date from Jan 2005 to Dec 2020, in Trials

A

Treatment	/	/	TAX/IRT	TAS-102	Everolimus	Regorafenib	Apatinib	Nivolumab	Avelumab	Placebo
Overall	/	OS	0.68	0.58	0.18	0.48	**0.78**	0.77	0.49	0.03
	/	PFS	NA	0.51	0.28	0.86	**0.92**	0.43	NA	0.00
	/	DCR	NA	0.77	0.47	NA	**0.97**	0.29	NA	0.00
	/	⩾3AEs	NA	**0.98**	NA	0.38	NA	0.62	NA	0.01
	1-Year OS rate		0.63	0.43	0.13	NA	0.71	**0.76**	0.74	0.10
	6-Months PFS rate		NA	0.48	0.64	NA	**0.82**	0.56	NA	0.00
HER-2	Positive	OS	NA	0.44	NA	NA	NA	**0.98**	NA	0.08
	Positive	PFS	NA	**0.77**	NA	NA	NA	0.73	NA	0.00
	Negative	OS	NA	**0.89**	NA	NA	NA	0.61	NA	0.00
	Negative	PFS	NA	**0.92**	NA	NA	NA	0.58	NA	0.00
Previous regimens	2	OS	0.49	**0.75**	0.33	NA	0.71	0.60	NA	0.12
	3	OS	NA	**0.70**	NA	NA	0.62	0.60	NA	0.08
Previous gastrectomy	Yes	OS	NA	**0.87**	0.20	NA	NA	0.79	NA	0.14
	No	OS	NA	0.63	0.45	NA	NA	**0.84**	NA	0.07
Gender	Male	OS	0.71	0.61	0.19	NA	NA	**0.73**	0.72	0.04
	Female	OS	**0.83**	0.59	0.37	NA	NA	0.66	0.29	0.26
Age	Age<65	OS	0.70	**0.78**	0.18	NA	0.61	0.72	0.37	0.15
	Age≥65	OS	0.69	0.42	0.26	NA	0.69	0.66	**0.74**	0.04
ECOG	PS=1	OS	0.63	0.71	0.34	NA	0.40	**0.76**	0.60	0.07
	PS=0	OS	0.76	0.62	0.08	NA	**0.81**	0.70	0.34	0.19
Histological type	Diffuse	OS	NA	**0.75**	0.33	NA	NA	0.72	NA	0.20
	Intestinal	OS	NA	**0.86**	0.18	NA	NA	0.79	NA	0.18
Primary sites	GEJ	OS	NA	0.56	0.41	NA	NA	**0.95**	NA	0.08
	Gastric	OS	NA	**0.85**	0.24	NA	NA	0.80	NA	0.11
Metastasis site	1	OS	**0.83**	0.60	NA	NA	0.56	0.49	NA	0.02
	2	OS	0.69	0.51	NA	NA	0.48	**0.78**	NA	0.04
Measurable lesion	Yes	OS	0.26	0.72	NA	NA	NA	**0.95**	NA	0.08
	No	OS	0.56	**0.91**	NA	NA	NA	0.50	NA	0.03

B

Treatment	/	/	TAX/IRT	TAS-102	Everolimus	Regorafenib	Apatinib	Nivolumab	Avelumab	Placebo
Overall	/	OS	3	4	7	6	1	2	5	8
	/	PFS	NA	3	5	2	1	4	NA	6
	/	DCR	NA	2	3	NA	1	4	NA	5
	/	⩾3AEs	NA	1	NA	3	NA	2	NA	4
	1-Year OS rate		4	5	6	NA	3	1	2	7
	6-Months PFS rate		NA	4	2	NA	1	NA	3	5
HER-2	Positive	OS	NA	2	NA	NA	NA	1	NA	3
	Positive	PFS	NA	1	NA	NA	NA	2	NA	3
	Negative	OS	NA	1	NA	NA	NA	2	NA	3
	Negative	PFS	NA	1	NA	NA	NA	2	NA	3
Previous regimens	2	OS	4	1	5	NA	2	3	NA	6
	3	OS	NA	1	NA	NA	2	3	NA	4
Previous gastrectomy	Yes	OS	NA	1	3	NA	NA	2	NA	4
	No	OS	NA	2	3	NA	NA	1	NA	4
Gender	Male	OS	3	4	5	NA	NA	1	2	6
	Female	OS	1	3	4	NA	NA	2	5	6
Age	Age<65	OS	3	1	6	NA	4	2	5	7
	Age≥65	OS	2	5	6	NA	3	4	1	7
ECOG	PS=1	OS	3	2	6	NA	5	1	4	7
	PS=0	OS	2	4	7	NA	1	3	5	6
Histological type	Diffuse	OS	NA	1	3	NA	NA	2	NA	4
	Intestinal	OS	NA	1	4	NA	NA	2	NA	3
Primary sites	GEJ	OS	NA	2	3	NA	NA	1	NA	4
	Gastric	OS	NA	1	3	NA	NA	2	NA	4
Metastasis site	1	OS	1	2	NA	NA	3	4	NA	5
	2	OS	2	3	NA	NA	4	1	NA	5
Measurable lesion	Yes	OS	3	2	NA	NA	NA	1	NA	4
	No	OS	2	1	NA	NA	NA	3	NA	4

C

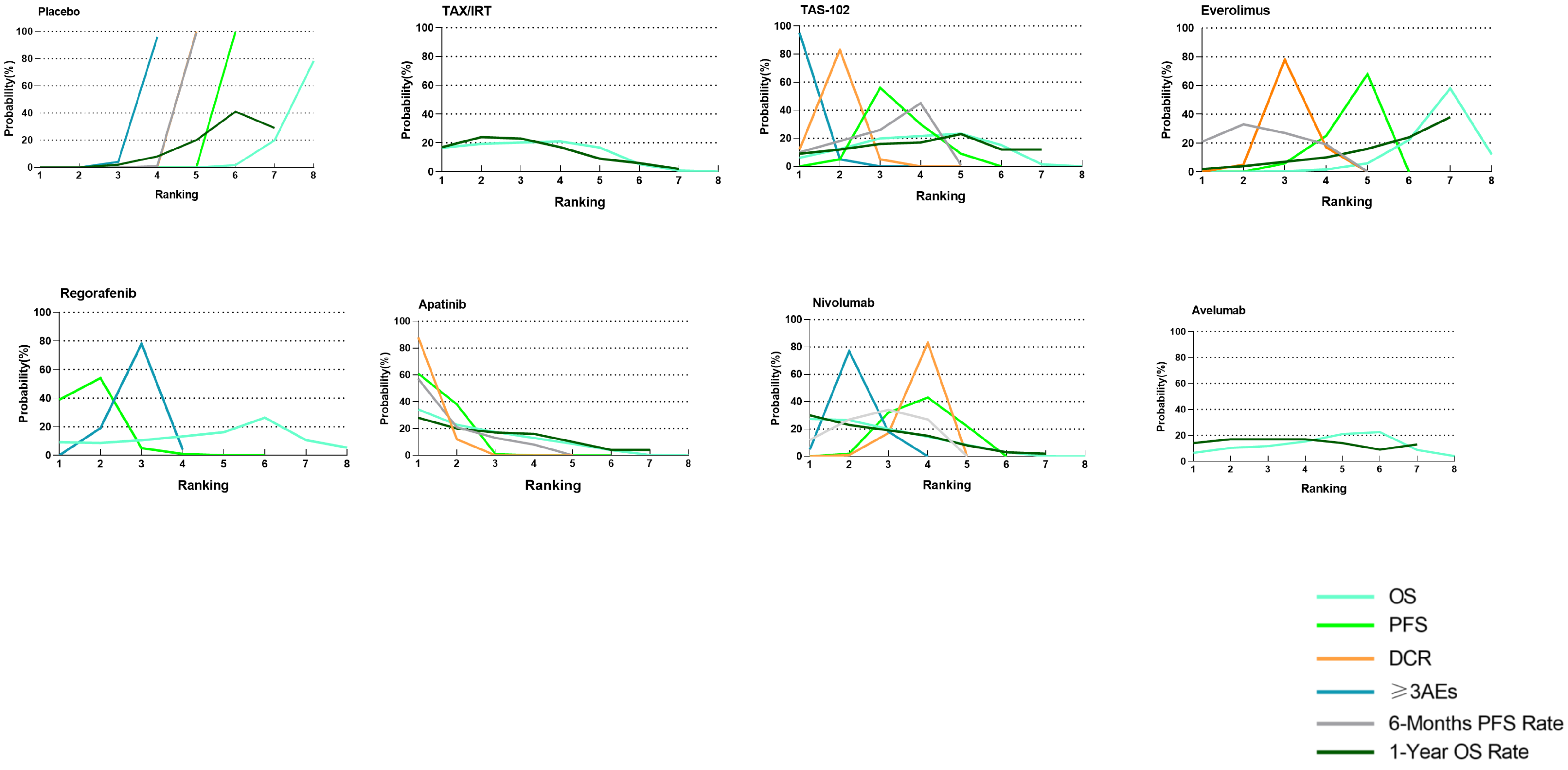
Placebo
TAX/IRT
TAS-102
Everolimus
Regorafenib
Apatinib
Nivolumab
Avelumab
Probability(%)
Ranking
OS
PFS
DCR
⩾3AEs
6-Months PFS Rate
1-Year OS Rate

D

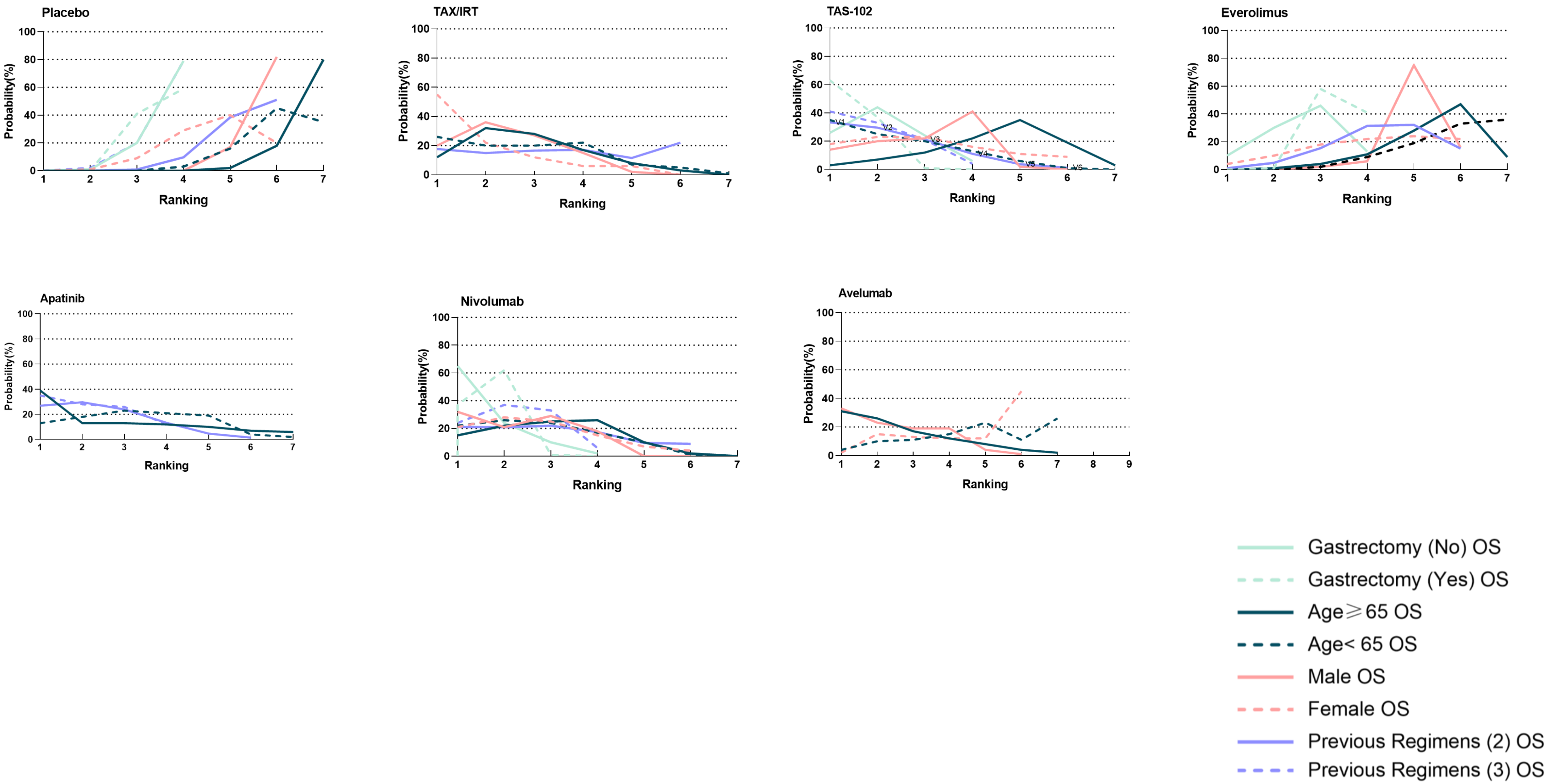
Placebo
TAX/IRT
TAS-102
Everolimus
Apatinib
Nivolumab
Avelumab
Probability(%)
Ranking
Gastrectomy (No) OS
Gastrectomy (Yes) OS
Age⩾65 OS
Age< 65 OS
Male OS
Female OS
Previous Regimens (2) OS
Previous Regimens (3) OS

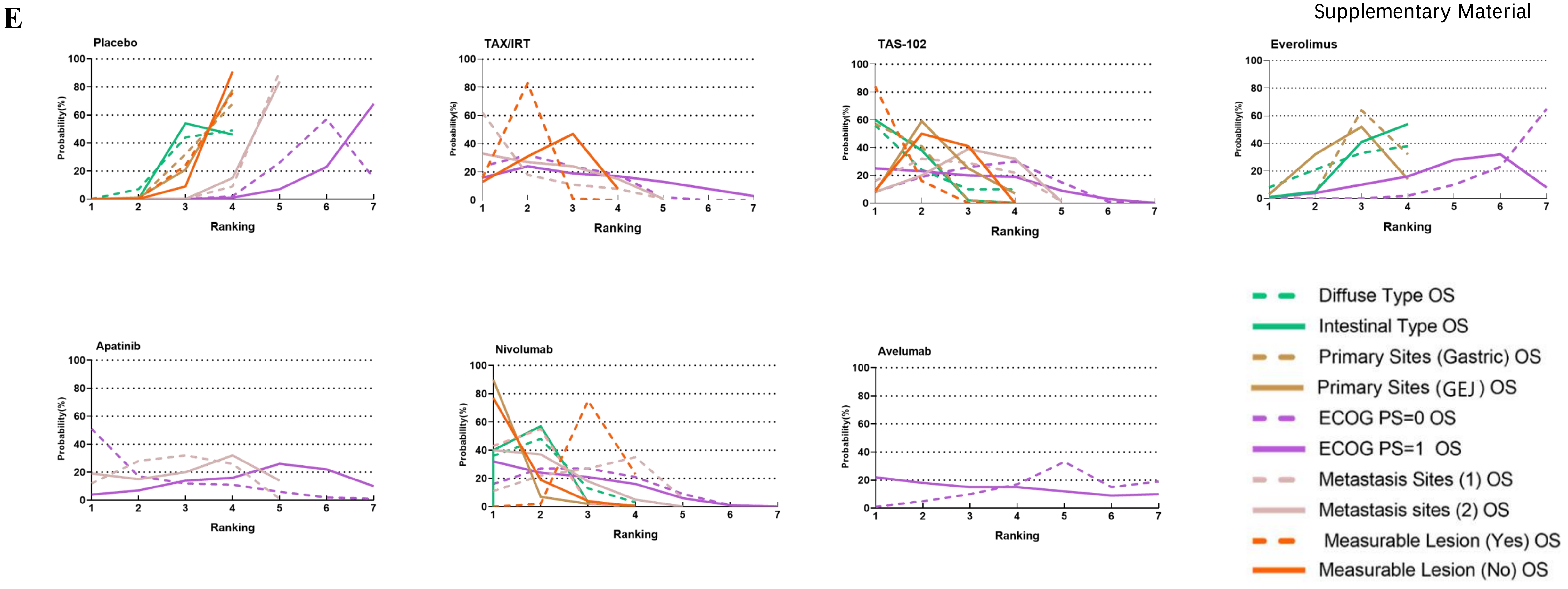

Supplementary Figure 1. Bayesian ranking profiles of comparable treatments on efficacy and safety for patients with advanced GC/GEJ cancer.
(A) Value of the surface under the cumulative ranking curve (SUCRA) in each cell indicates the probability of each treatment being ranked from first to last on overall survival (OS), progression free survival (PFS), OS subgroup, the probability of value being ranked first are bold. (B) Number in each cell indicate the probability of each treatment being ranked from first to last on OS, PFS, OS subgroup according to the value of surface under the cumulative ranking curve (SUCRA). (B) Value of the surface under the cumulative ranking curve (SUCRA) in each cell indicates the probability of each treatment being ranked from first to last on OS, PFS, OS subgroup, the probability of value being ranked first are bold. (C) Profiles indicate the probability of each comparable treatment being ranked from first to last on OS, PFS, disease control rate (DCR), adverse events of grade 3 or higher (≥ 3AEs), 1-year OS rate, 6-months PFS rate. (D) Profiles indicate the probability of each comparable treatment being ranked from first to last on number of previous regimens (2, 3), previous gastrectomy (Yes, No), female and male, age ≥65 and <65. (E) Profiles indicate the probability of each comparable treatment being ranked from first to last on Eastern Cooperative Oncology Group Performance Status=0/1 (ECOG PS=0, ECOG PS=1), primary sites (Gastric, Gastroesophageal junction), metastasis sites (1, 2), measurable lesion (Yes, No). Abbreviations: TAX, Paclitaxel/Docetaxel; IRT, Irinotecan; HER-2, human epidermal growth factor receptor-2; ECOG PS, Eastern Cooperative Oncology Group Performance Status; GC/GEJ cancer, Gastric cancer/ Gastroesophageal junction cancer.

Supplementary Figure 2. A frequency toxicity profile in relation to the incidence (%) of each ≥3 adverse events based on the population of each treatment in NMA we included.

	NO. of studies	Sample size	TAX/IRT	TAS-102	Everolimus	Regorafenib	Apatinib	Nivolumab	Avelumab	Placebo
**Neutropenia**	6	2112	2.4	5.4	0.8	NA	0.6	NA	0.0	1.7
**Leukopenia**	4	1272	NA	2.4	NA	NA	0.4	NA	0.0	0.9
**Thrombocytopenia**	5	1751	0.2	0.6	1.3	NA	0.4	NA	NA	0.5
**Anaemia**	8	2593	1.9	2.5	2.7	NA	0.5	1.5	0.0	3.5
**Appetite Decreased**	5	2268	NA	1.3	2.1	NA	0.2	0.2	0.0	1.3
**Nausea**	6	2405	NA	0.5	0.7	NA	0.2	0.0	0.0	0.7
**Vomiting**	6	2326	0.3	0.5	0.6	NA	0.0	0.2	0.0	0.9
**Diarrhoea**	7	2603	0.6	0.3	0.6	NA	0.0	0.1	0.0	0.7
**Abdominal Pain**	6	2201	NA	0.6	1.0	0.2	0.1	0.6	NA	1.8
**Constipation**	3	1646	NA	0.2	0.2	NA	NA	0.1	NA	0.4
**AST Increased**	3	999	NA	NA	NA	0.9	NA	0.2	0.4	0.4
**ALT Increased**	3	999	NA	NA	NA	0.8	NA	0.1	0.3	0.8
**Fatigue**	7	2603	1.0	0.9	1.3	NA	2.0	0.1	0.0	1.7
**Asthenia**	4	2007	NA	0.8	1.0	NA	NA	0.5	0.0	1.3
**Fever**	3	1284	NA	NA	0.2	NA	0.0	0.1	NA	0.2
**Dyspnea**	3	1646	NA	0.4	1.1	NA	NA	0.1	NA	0.8
**Hypertension**	3	555	NA	NA	NA	1.8	1.4	NA	NA	1.0
**Proteinuria**	2	408	NA	NA	NA	NA	0.9	NA	NA	0.5
**Back Pain**	3	1646	NA	0.1	0.6	NA	NA	0.1	NA	0.5
**Hand-foot syndrome**	2	408	NA	NA	NA	NA	3.9	NA	NA	1.5

Abbreviations: NMA, Network meta-analysis; NA, Not available; TAX, Docetaxel/Paclitaxel; IRT, Irinotecan; TAS-102, Trifluridine/Tipiracil.

A

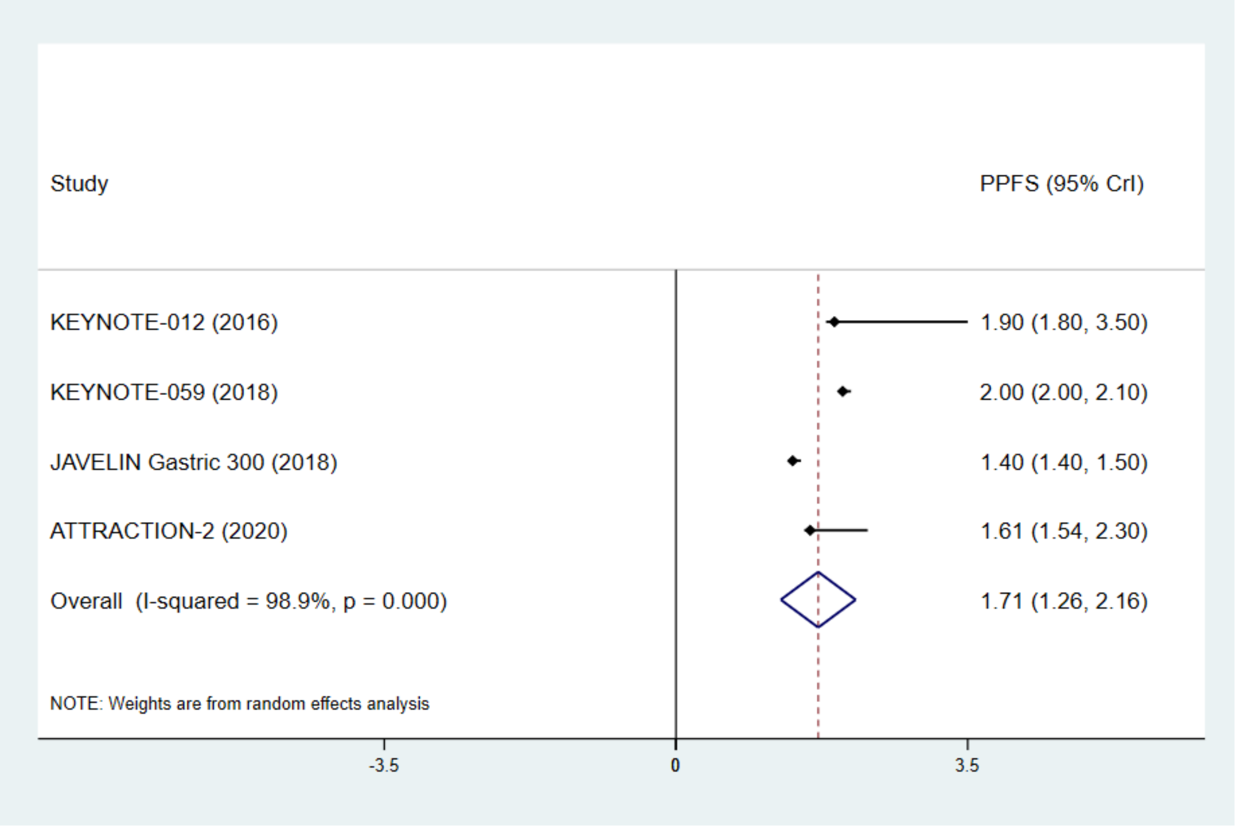

PPFS

B

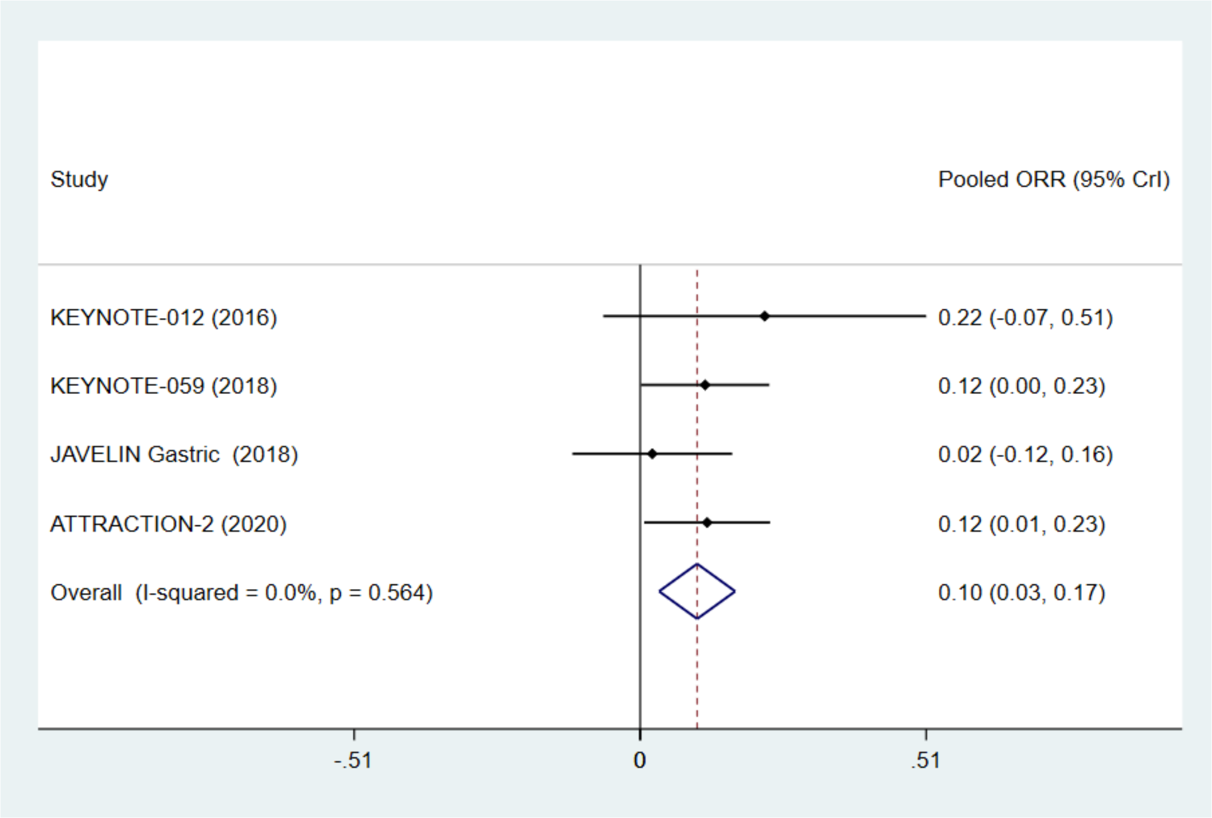

Pooled ORR

C

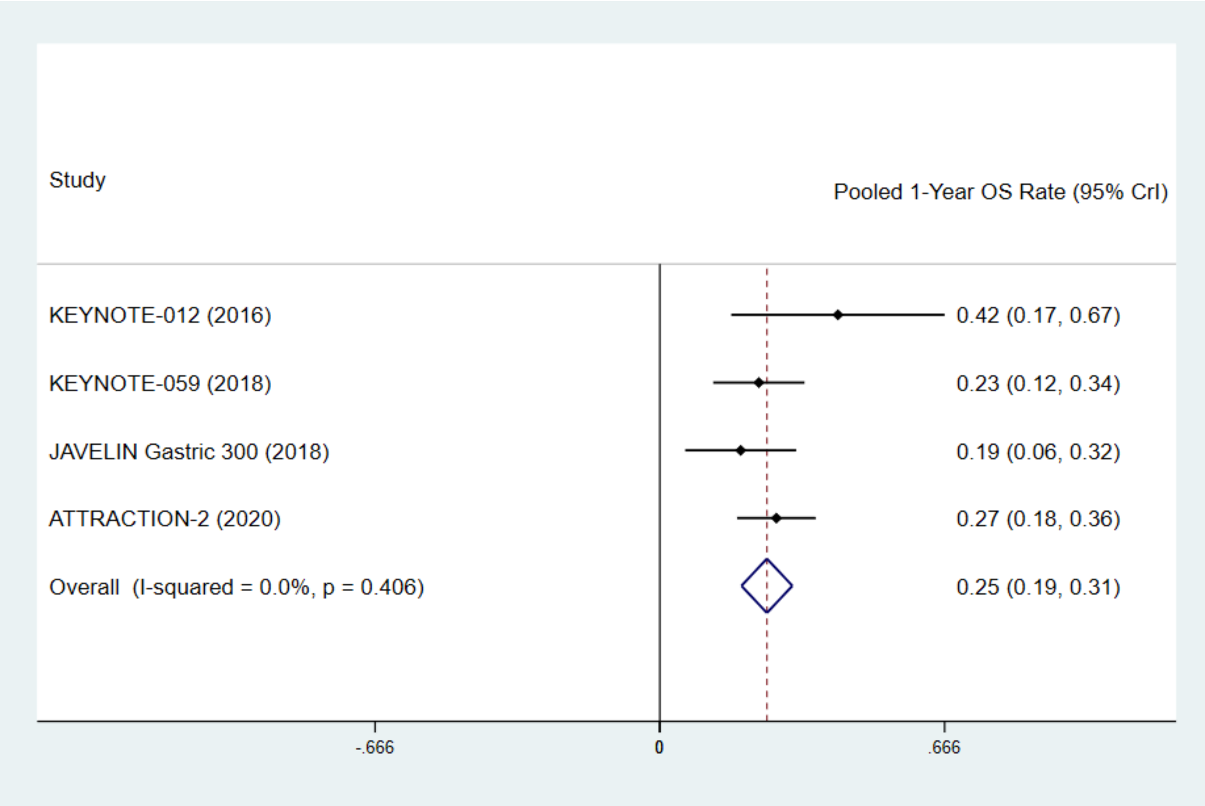

Pooled 1-Year OS Rate

D

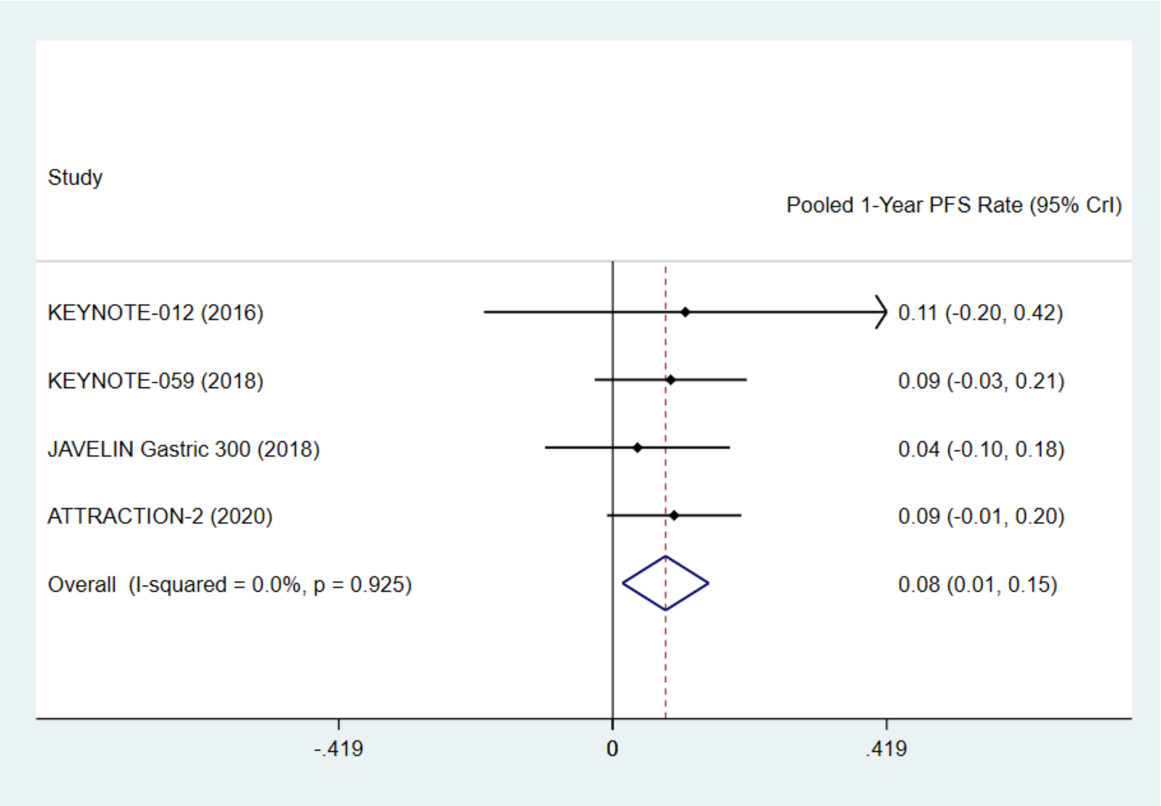

Pooled 1-Year PFS Rate

E

Any grade TRAEs of immune checkpoint inhibitors

	NO. of studies	Sample size	Nivolumab	Avelumab	Pembrolizumab
Anaemia	2	19	NA	0.5	0.9
Decreased appetite	4	46	0.3	0.1	0.5
Nausea	3	44	0.3	0.2	0.4
Diarrhoea	3	51	0.5	0.2	0.3
AST Increased	3	25	0.4	0.2	0.3
ALT Increased	2	14	0.5	0.5	NA
Fatigue	4	85	0.2	0.1	0.7
Pruritus	2	53	0.6	NA	0.4
Rash	2	41	0.5	NA	0.5
Hypothyroidism	3	34	0.3	NA	0.7
Arthralgia	2	19	NA	NA	1

F

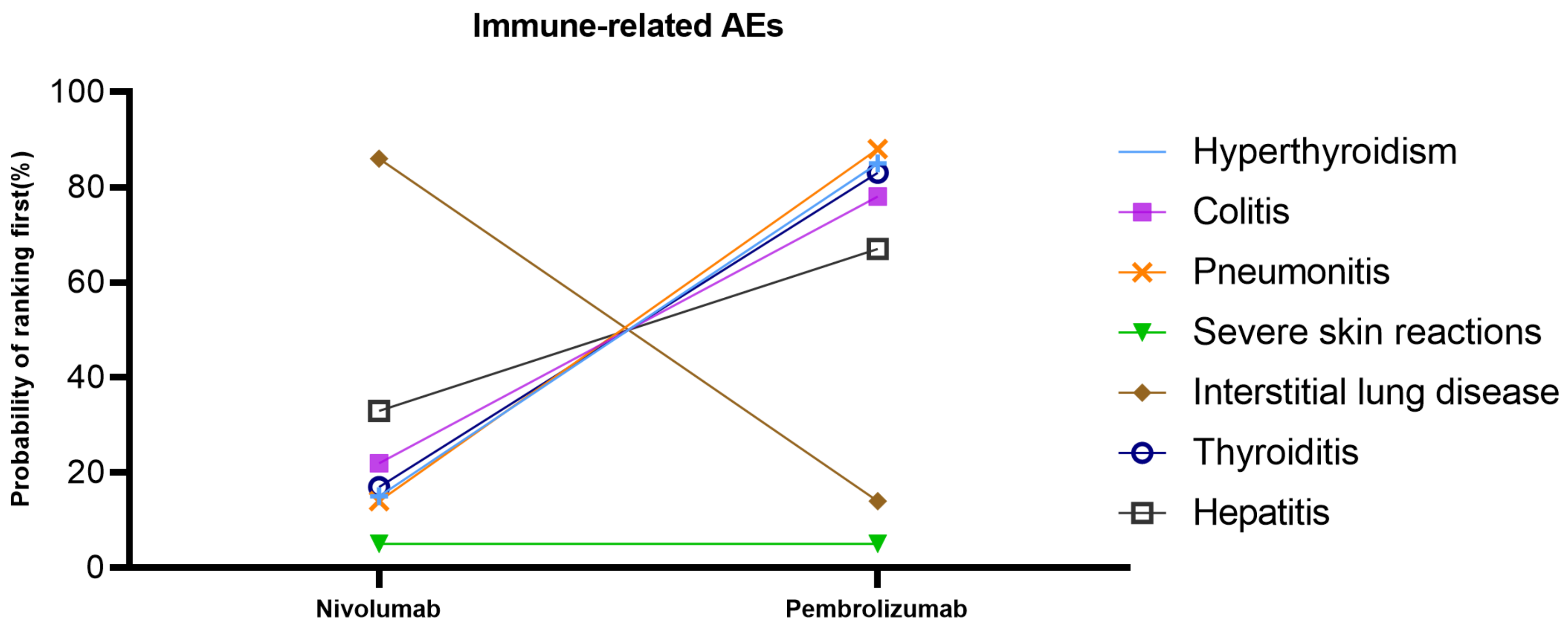

Supplementary Figure 3. Pooled survival outcomes from integrated analysis of median progression free survival (mPFS) (A), objective response rate (ORR) (B), 1-year OS rate (C), 1-year PFS rate (D) of immune checkpoint inhibitors in patients with for advanced GC/GEJ cancer. (E) A frequency toxicity profile in relation to the incidence of any grade treatment- related adverse event (TRAE) based on the population of each immune checkpoint inhibitor we included. (F) Relative toxicity of comparable treatments on seven common immune-related adverse event (irAE) of any grade.

Abbreviations: GC/GEJ cancer, Gastric cancer/ Gastroesophageal junction cancer.

A

≥3AEs

DCR

**TAS-102**		**3.95 (1.67, 9.40)**		2.43 (0.85, 6.25)	**7.46 (4.61, 12.51)**
1.74 (0.90, 3.40)	**Everolimus**				
		**Regorafenib**		0.61 (0.19, 1.77)	1.89 (0.93, 3.83)
0.60 (0.25, 1.40)	**0.35 (0.15, 0.74)**		**Apatinib**		
**2.34 (1.18, 4.76)**	1.35 (0.73, 2.49)		**3.89 (1.75, 9.22)**	**Nivolumab**	**3.07 (1.40, 7.80)**
**4.76 (2.86, 8.12)**	**2.72 (1.84, 4.09)**		**7.84 (4.12, 16.50)**	**2.02 (1.27, 3.25)**	**Placebo**

B

Diffuse type

Intestinal type

**TAS-102**	0.73 (0.33, 1.59)	0.92 (0.45, 1.90)	0.69 (0.36, 1.32)
0.57 (0.32, 1.02)	**Everolimus**	1.27 (0.73, 2.18)	0.95 (0.62, 1.46)
0.94 (0.55, 1.58)	1.64 (0.96, 2.81)	**Nivolumab**	0.75 (0.54, 1.05)
**0.58 (0.39, 0.87)**	1.02 (0.67, 1.55)	**0.62 (0.44, 0.87)**	**Placebo**

Histological type (OS)

C

No

Yes

**TAS-102**	0.92 (0.63, 1.35)	1.13 (0.73, 1.74)	0.80 (0.60, 1.06)
**0.59 (0.39, 0.89)**	**Everolimus**	1.22 (0.81, 1.86)	0.87 (0.68, 1.12)
0.93 (0.62, 1.41)	**1.59 (1.10, 2.29)**	**Nivolumab**	**0.71 (0.51, 0.99)**
**0.57 (0.41, 0.79)**	0.97 (0.75, 1.26)	**0.61 (0.47, 0.79)**	**Placebo**

Previous gastrectomy (OS)

D

Gastroesophageal junction

Gastric

**TAS-102**	0.89 (0.53, 1.49)	1.78 (0.77, 4.17)	0.75 (0.50, 1.12)
**0.71 (0.50, 0.99)**	**Everolimus**	2.00 (0.89, 4.50)	0.84 (0.61, 1.16)
0.97 (0.69, 1.36)	**1.38 (1.01, 1.87)**	**Nivolumab**	**0.42 (0.20, 0.89)**
**0.67 (0.52, 0.87)**	0.95 (0.76, 1.18)	**0.69 (0.56, 0.86)**	**Placebo**

Primary sites (OS)

E

Age<65

Age⩾65

**TAX/IRT**	1.06 (0.63, 1.79)	0.72 (0.44, 1.19)	0.95 (0.56, 1.63)	1.02 (0.61, 1.70)	0.82 (0.60, 1.12)	0.71 (0.46, 1.11)
0.78 (0.45, 1.35)	**TAS-102**	**0.68 (0.47, 0.98)**	0.89 (0.59, 1.35)	0.96 (0.65, 1.40)	0.77 (0.42, 1.41)	**0.67 (0.51, 0.89)**
0.69 (0.41, 1.15)	0.88 (0.57, 1.36)	**Everolimus**	1.32 (0.89, 1.95)	**1.41 (1.00, 2.01)**	1.14 (0.63, 2.05)	0.99 (0.78, 1.25)
1.04 (0.43, 2.49)	1.33 (0.58, 3.05)	1.51 (0.67, 3.39)	**Apatinib**	1.07 (0.72, 1.61)	0.86 (0.46, 1.60)	0.75 (0.55, 1.02)
0.95 (0.56, 1.62)	1.22 (0.77, 1.93)	1.38 (0.91, 2.10)	0.92 (0.40, 2.08)	**Nivolumab**	0.80 (0.44, 1.46)	**0.70 (0.54, 0.91)**
1.05 (0.72, 1.53)	1.34 (0.69, 2.61)	1.53 (0.81, 2.89)	1.01 (0.39, 2.63)	1.10 (0.58, 2.12)	**Avelumab**	0.87 (0.51, 1.49)
**0.57 (0.37, 0.88)**	0.73 (0.52, 1.02)	0.83 (0.63, 1.10)	0.55 (0.26, 1.18)	**0.60 (0.44, 0.82)**	**0.54 (0.31, 0.96)**	**Placebo**

OS

F

Female

Male

**TAX/IRT**	0.82 (0.40, 1.66)	0.70 (0.36, 1.36)	0.85 (0.44, 1.66)	0.65 (0.42, 1.01)	0.67 (0.38, 1.17)
0.92 (0.59, 1.45)	**TAS-102**	0.85 (0.49, 1.50)	1.04 (0.59, 1.84)	0.79 (0.34, 1.82)	0.82 (0.53, 1.27)
0.67 (0.44, 1.02)	**0.72 (0.52, 1.00)**	**Everolimus**	1.21 (0.73, 2.03)	0.93 (0.42, 2.06)	0.96 (0.67, 1.37)
1.00 (0.65, 1.55)	1.08 (0.77, 1.53)	**1.50 (1.10, 2.05)**	**Nivolumab**	0.76 (0.34, 1.70)	0.79 (0.55, 1.14)
1.01 (0.76, 1.34)	1.09 (0.64, 1.86)	1.51 (0.91, 2.52)	1.01 (0.60, 1.70)	**Avelumab**	1.03 (0.51, 2.10)
**0.60 (0.41, 0.87)**	**0.65 (0.50, 0.84)**	0.90 (0.73, 1.11)	**0.60 (0.48, 0.75)**	**0.60 (0.37, 0.95)**	**Placebo**

OS

G

ECOG PS=1

ECOG PS=0

**TAX/IRT**	1.04 (0.62, 1.75)	0.83 (0.50, 1.38)	0.86 (0.50, 1.49)	1.07 (0.64, 1.77)	0.99 (0.74, 1.32)	0.72 (0.46, 1.12)
0.88 (0.50, 1.53)	**TAS-102**	0.80 (0.57, 1.13)	0.83 (0.56, 1.24)	1.03 (0.73, 1.46)	0.95 (0.53, 1.72)	**0.69 (0.53, 0.89)**
**0.52 (0.30, 0.89)**	**0.59 (0.36, 0.97)**	**Everolimus**	1.04 (0.70, 1.52)	1.28 (0.93, 1.78)	1.19 (0.66, 2.13)	0.86 (0.68, 1.08)
1.13 (0.51, 2.52)	1.29 (0.60, 2.78)	**2.19 (1.02, 4.70)**	**Apatinib**	1.24 (0.84, 1.82)	1.15 (0.62, 2.12)	0.83 (0.61, 1.13)
0.95 (0.54, 1.66)	1.08 (0.65, 1.80)	**1.84 (1.11, 3.03)**	0.84 (0.39, 1.82)	**Nivolumab**	0.93 (0.52, 1.66)	**0.67 (0.53, 0.84)**
0.68 (0.44, 1.03)	0.77 (0.38, 1.56)	1.31 (0.66, 2.62)	0.6 (0.24, 1.49)	0.71 (0.35, 1.44)	**Avelumab**	0.72 (0.42, 1.23)
**0.59 (0.38, 0.90)**	**0.67 (0.47, 0.96)**	1.14 (0.81, 1.61)	0.52 (0.26, 1.03)	**0.62 (0.43, 0.89)**	0.87 (0.48, 1.58)	**Placebo**

OS

H

Three

Two

**TAX/IRT**					
1.19 (0.60, 2.39)	**TAS-102**		0.96 (0.53, 1.73)	0.94 (0.58, 1.52)	0.73 (0.50, 1.06)
0.90 (0.48, 1.71)	0.76 (0.49, 1.17)	**Everolimus**			
1.16 (0.58, 2.31)	0.97 (0.59, 1.61)	1.29 (0.84, 1.98)	**Apatinib**	0.98 (0.56, 1.70)	0.76 (0.48, 1.20)
1.08 (0.51, 2.30)	0.91 (0.50, 1.64)	1.20 (0.71, 2.04)	0.93 (0.52, 1.68)	**Nivolumab**	0.78 (0.57, 1.07)
0.81 (0.45, 1.46)	**0.68 (0.47, 0.98)**	0.90 (0.70, 1.15)	**0.70 (0.49, 0.99)**	0.75 (0.47, 1.20)	**Placebo**

Number of previous regimens (OS)

I

Two (upper triangle); One (lower triangle)

**TAX/IRT**	0.89 (0.55, 1.44)	0.86 (0.43, 1.73)	1.05 (0.66, 1.66)	**0.63 (0.42, 0.94)**
0.82 (0.44, 1.50)	**TAS-102**	0.97 (0.51, 1.84)	1.18 (0.83, 1.70)	**0.71 (0.54, 0.94)**
0.79 (0.43, 1.45)	0.97 (0.61, 1.54)	**Apatinib**	1.22 (0.66, 2.26)	0.73 (0.41, 1.29)
0.76 (0.40, 1.44)	0.93 (0.56, 1.56)	0.96 (0.58, 1.59)	**Nivolumab**	**0.60 (0.48, 0.76)**
**0.55 (0.33, 0.92)**	**0.68 (0.49, 0.95)**	**0.70 (0.51, 0.97)**	0.73 (0.49, 1.08)	**Placebo**

Metastasis sites (OS)

J

Yes (upper triangle); No (lower triangle)

**TAX/IRT**	1.04 (0.67, 1.61)	1.26 (0.82, 1.94)	0.77 (0.53, 1.12)
1.72 (0.59, 5.01)	**TAS-102**	1.21 (0.88, 1.66)	**0.74 (0.59, 0.93)**
**0.42 (0.20, 0.90)**	**0.25 (0.09, 0.66)**	**Nivolumab**	**0.61 (0.49, 0.76)**
**0.36 (0.20, 0.67)**	**0.21 (0.09, 0.50)**	0.85 (0.55, 1.32)	**Placebo**

Measurable lesion (OS)

Supplementary Figure 4. Network meta-analysis of the third-line treatments for advanced GC/GEJ cancer.
((A) Pooled odds ratio (OR) (95% CrI (credible interval)) for disease control rate (DCR) and adverse events of grade 3 or higher (≥3 AEs). (B) Pooled HR (95% CrI) for OS of histological type subgroup. (C) Pooled HR (95% CrI) for OS of previous gastrectomy (Yes, No). (D) Pooled HR (95% CrI) for OS of primary sites (Gastric, Gastroesophageal junction). (E) Pooled HR (95% CrI) for OS of age ≥ 65 and < 65. (F) Pooled HR (95% CrI) for OS of female and male. (G)Pooled HR (95% CrI) for OS of Eastern Cooperative Oncology Group Performance Status = 0/1 (ECOG PS=0, ECOG PS=1). (H) Pooled HR (95% CrI) for (OS) of number of previous regimens (two, three). (I) Pooled HR (95% CrI) for OS of metastasis sites(one, two). (J) Pooled HR (95% CrI) for OS of measurable lesion (Yes, No). Data in each cell are HR or OR (95% CrI) for the comparison of row-defining treatment versus column-defining treatment. HR less than 1 and OR more than 1 favour upper-row treatment. Significant results are highlighted in red and bold. Abbreviations: TAX, Paclitaxel/Docetaxel; IRT, Irinotecan; TAS-102, Trifluridine/Tipiracil; GC/GEJ cancer, Gastric cancer/ Gastroesophageal junction cancer.

Supplementary Figure 5. Summary of results from assessment of studies using the Cochrane risk of bias tool.

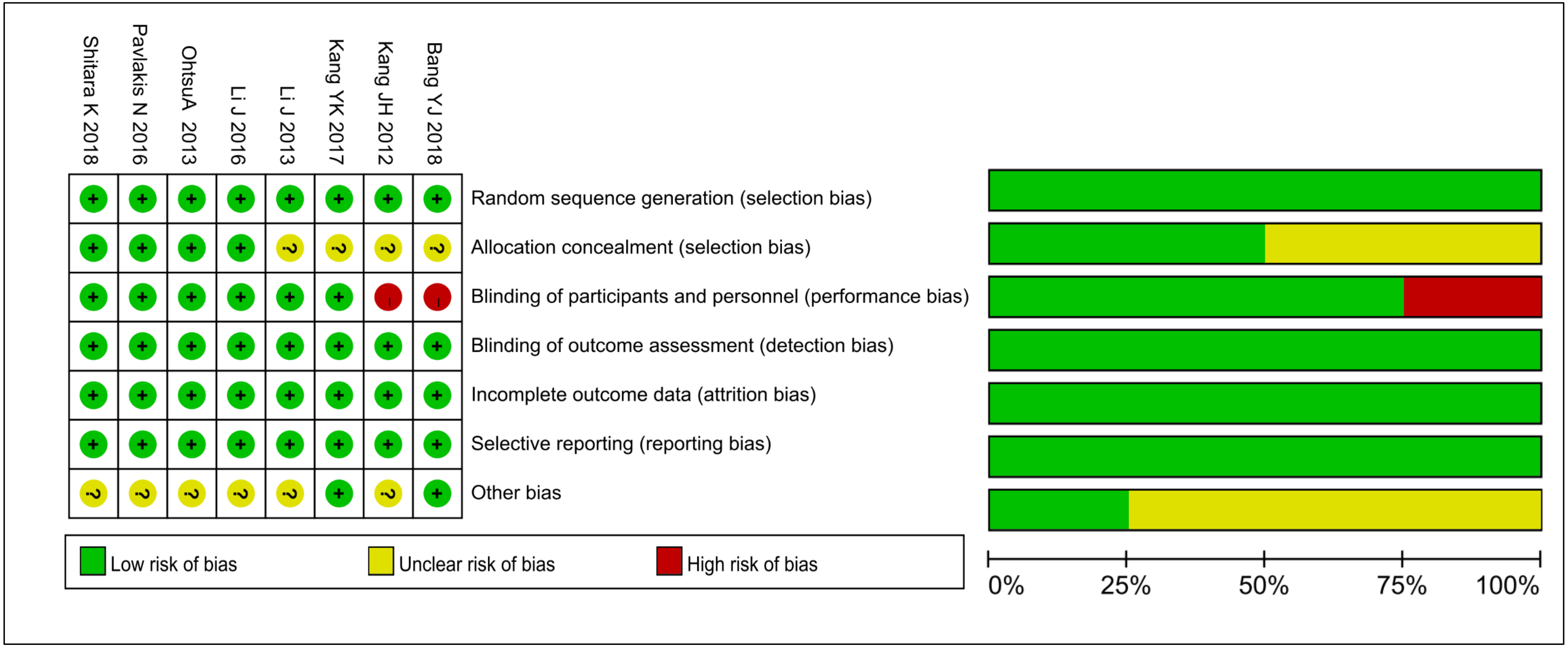

+: **low risk; ?: Unclear risk; - : high risk.**

OS

A. Brooks-Gelman-Rubin diagnostic

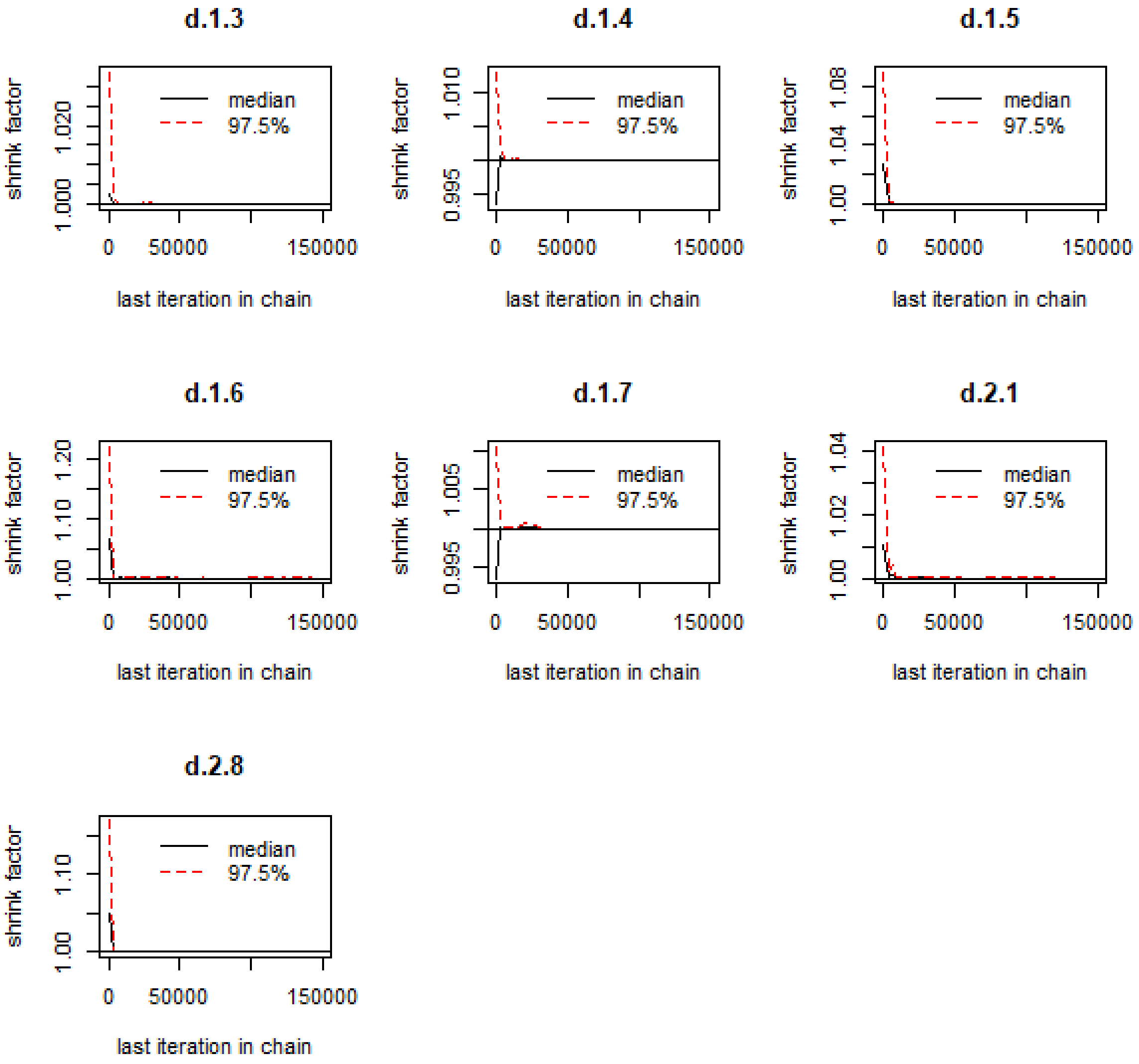

B. Trace plot

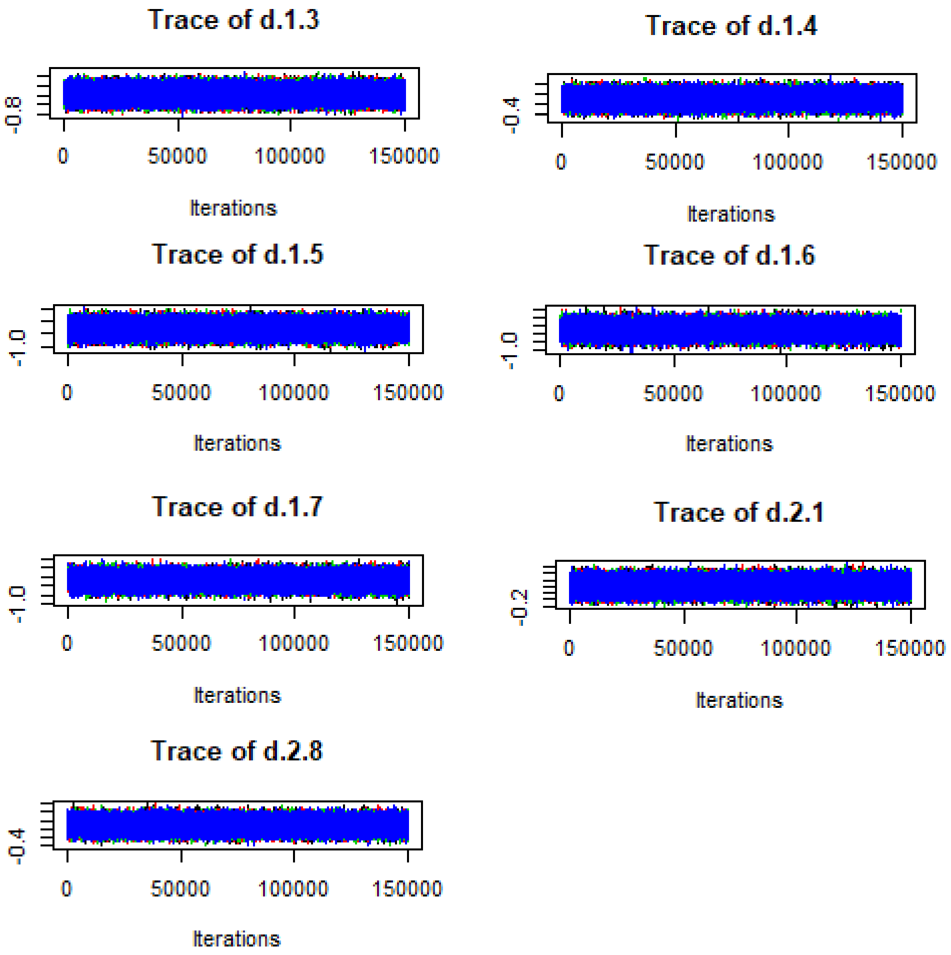

PFS

C. Brooks-Gelman-Rubin diagnostic

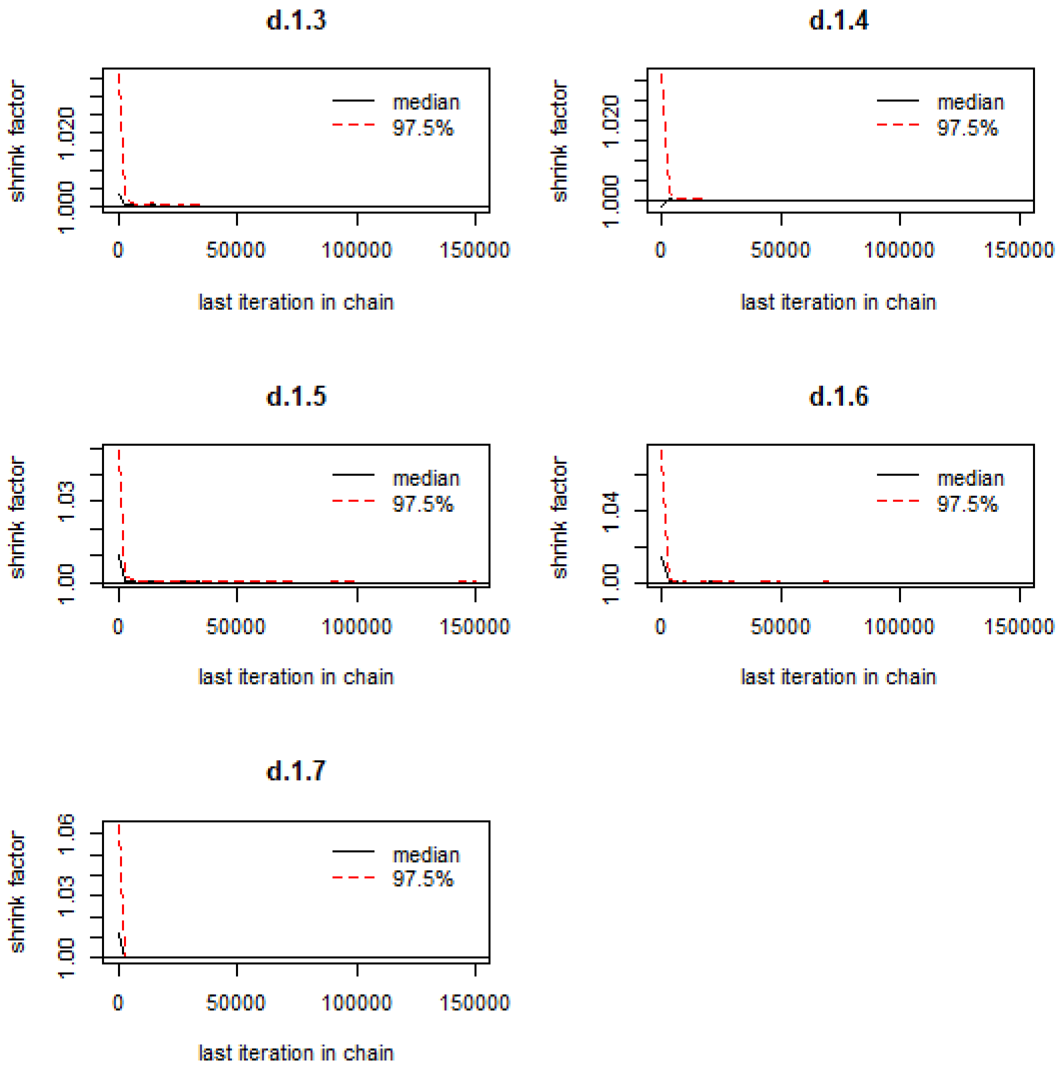

D. Trace plot

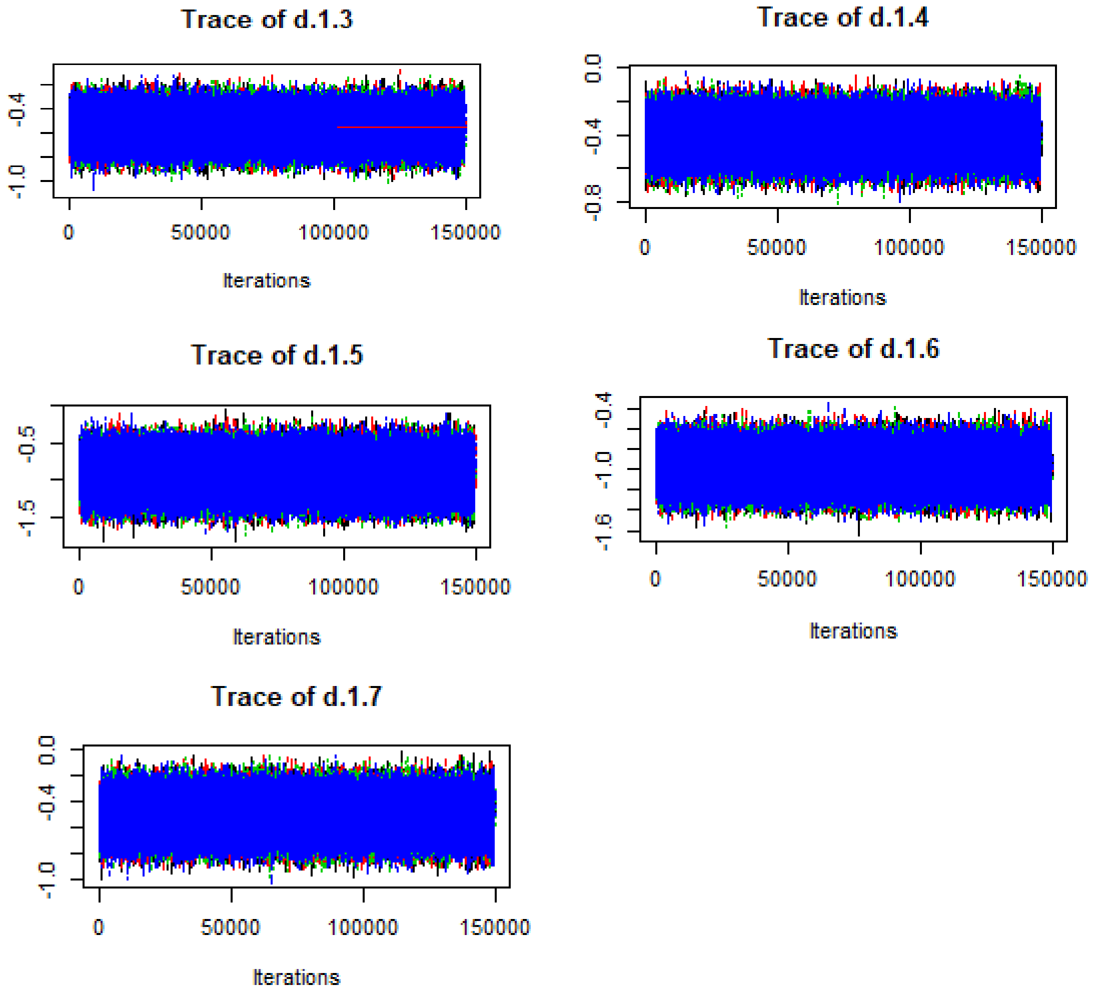

DCR

E. Brooks-Gelman-Rubin diagnostic

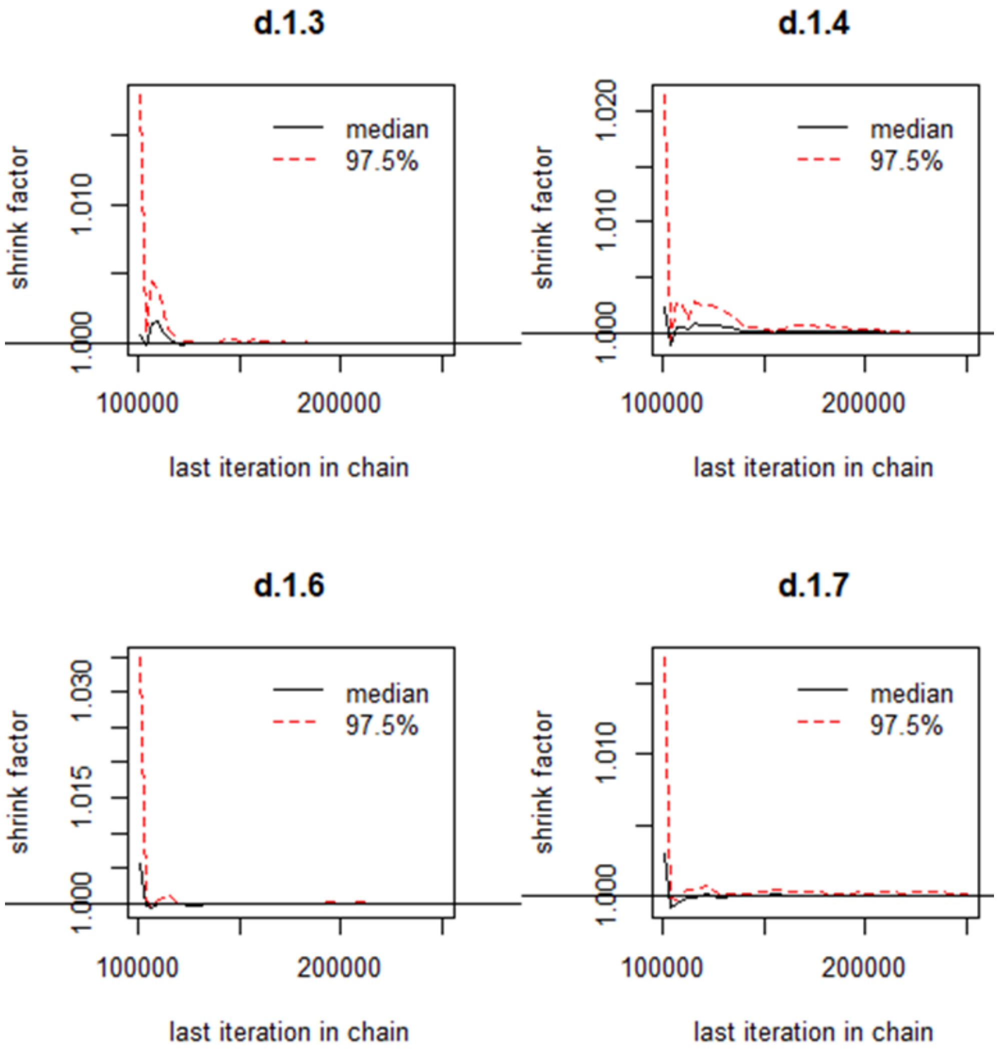

F. Trace plot

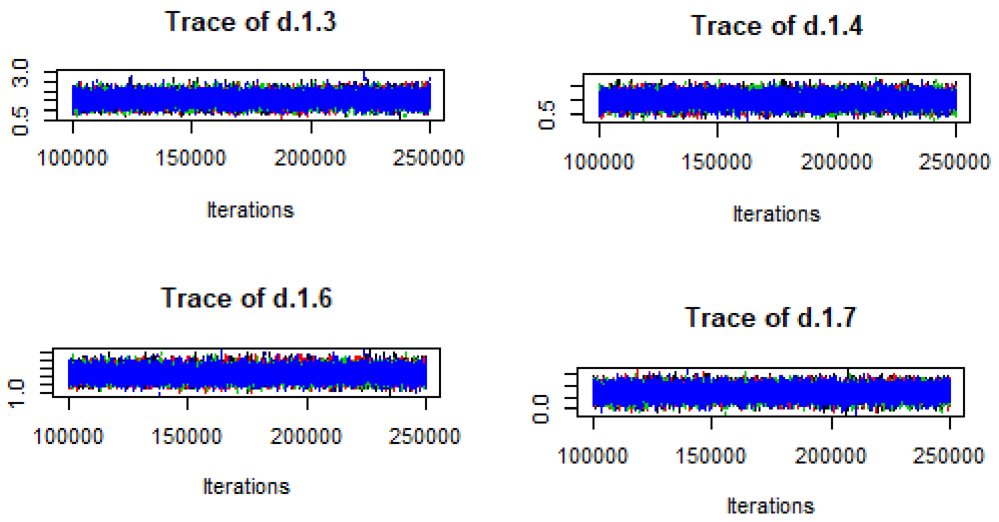

≥3 AEs

G. Brooks-Gelman-Rubin diagnostic

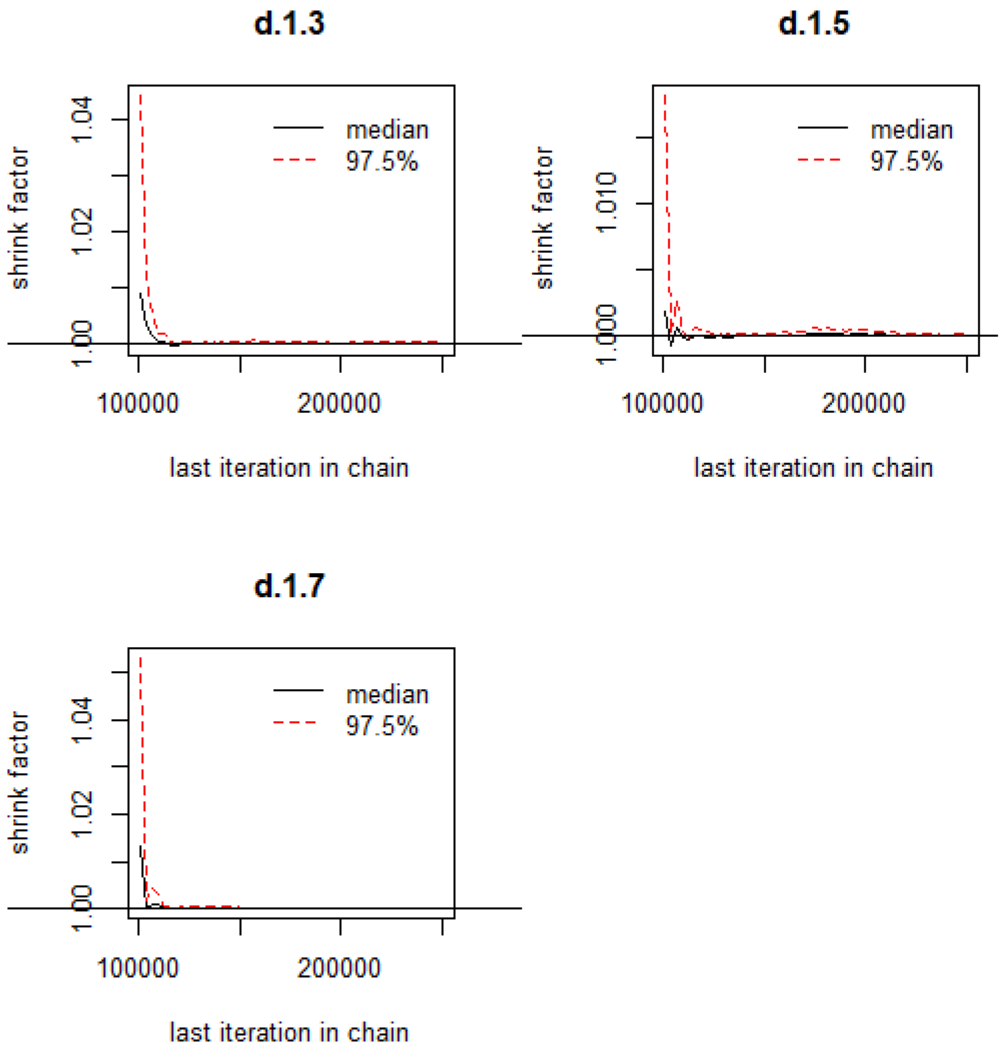

H. Trace plot

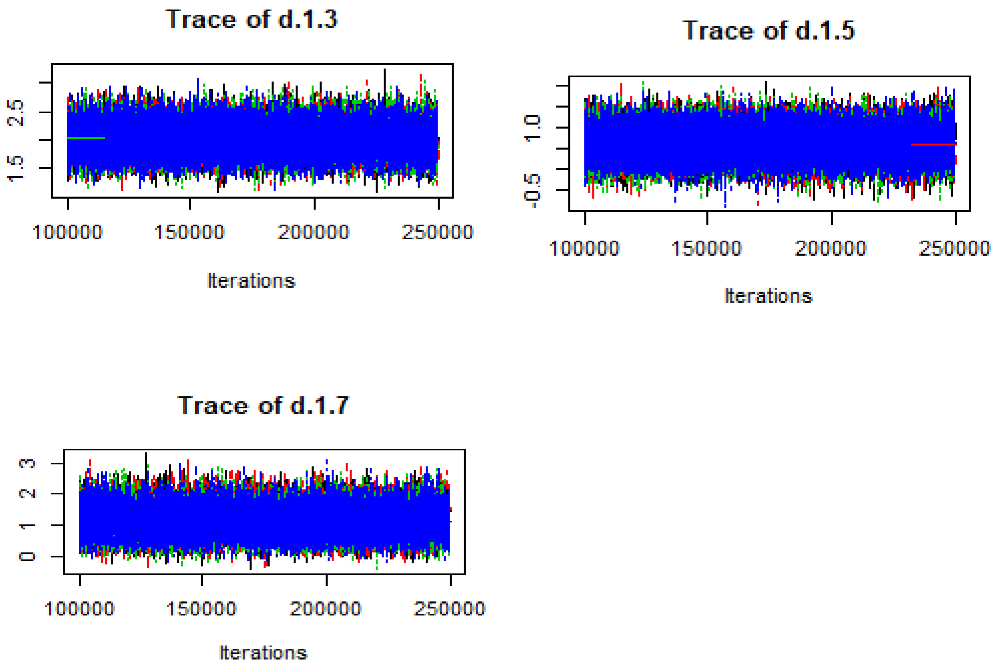

OS for ECOG PS=0

I. Brooks-Gelman-Rubin diagnostic

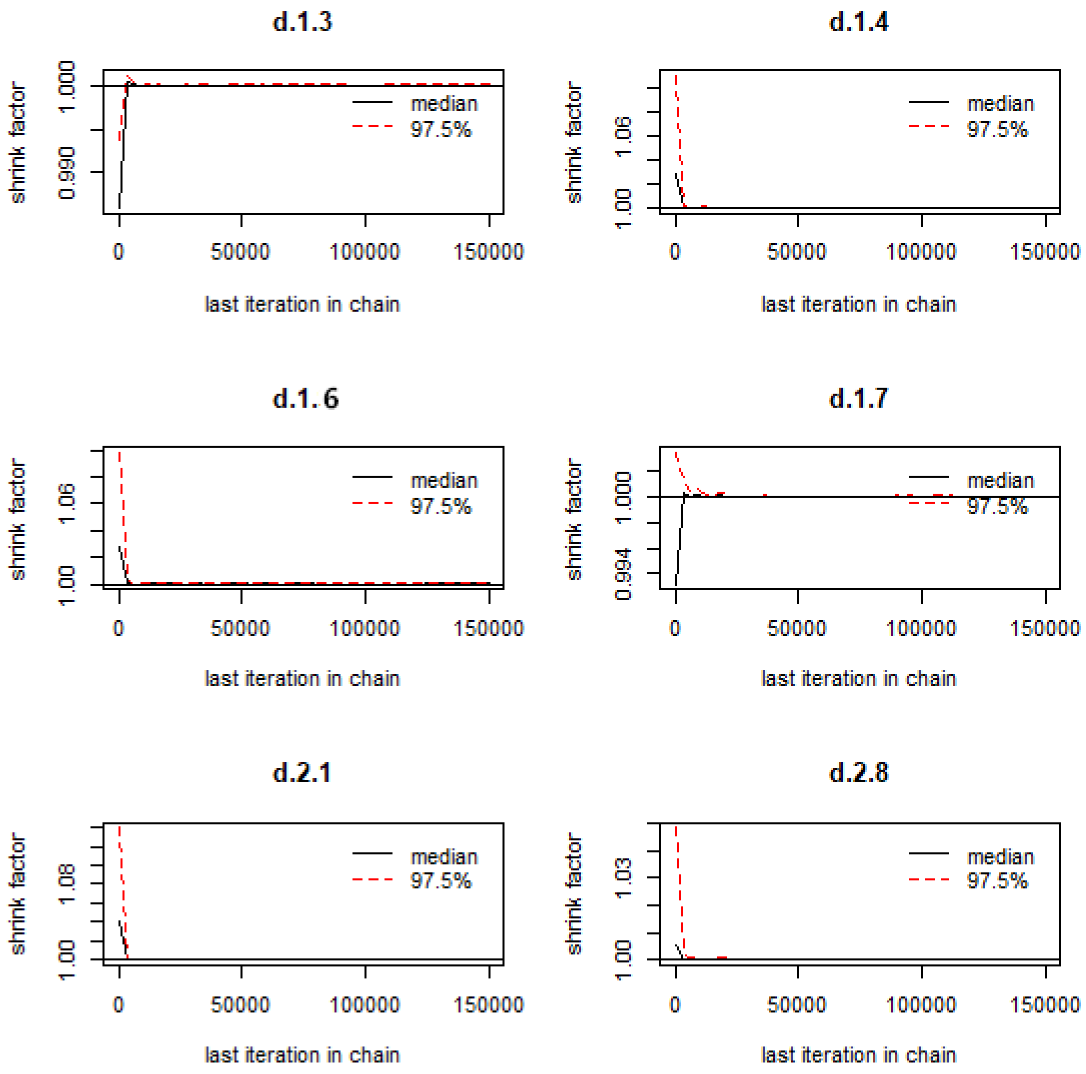

J. Trace plot

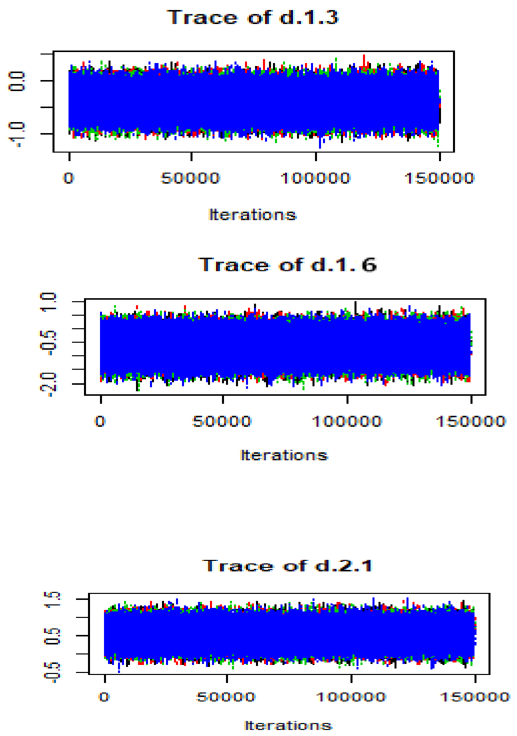

OS for ECOG PS=1

K. Brooks-Gelman-Rubin diagnostic

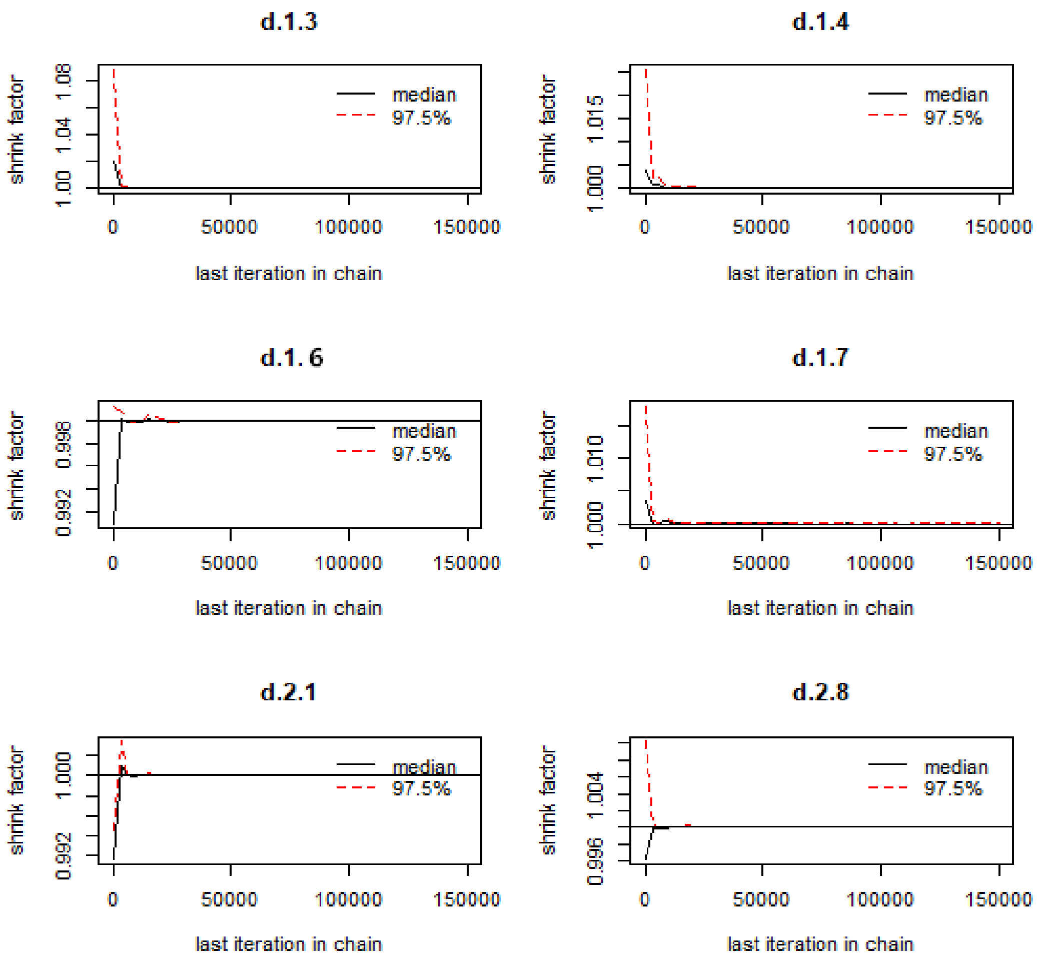

L. Trace plot

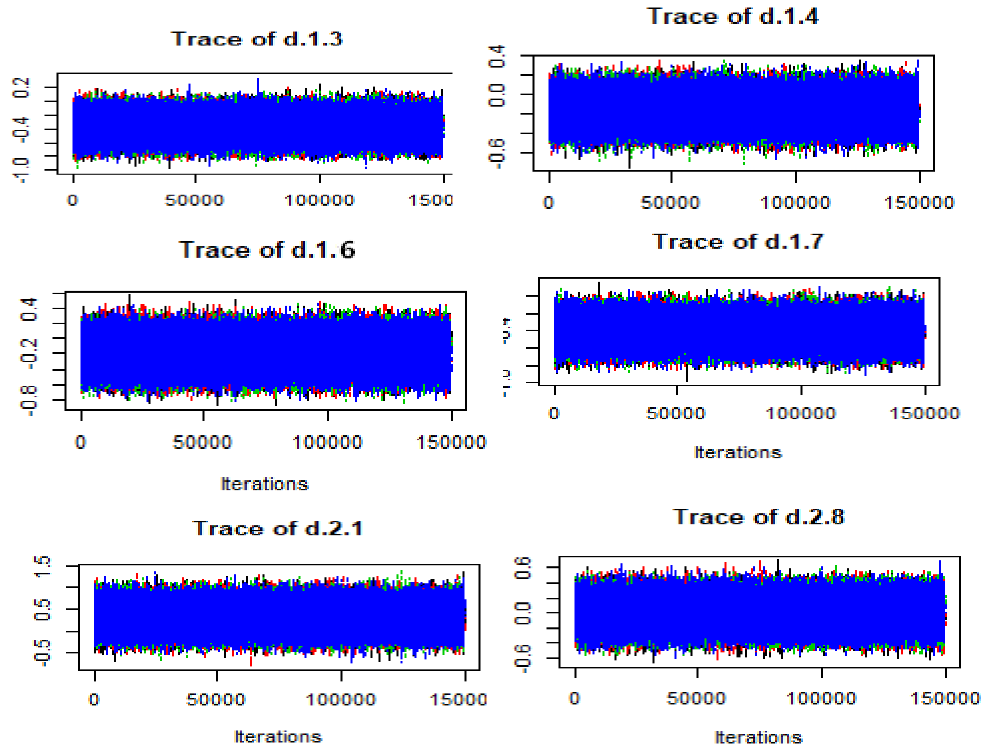

Supplementary Figure 6. Convergence of four chains established by the Brooks-Gelman-Rubin diagnostic and Trace plot and for overall survival (OS) (A and B), progression free survival (PFS) (C and D), disease control rate (DCR) (E and F), adverse events of grade 3 or higher (≥3 AEs) (G and H), OS for Eastern Cooperative Oncology Group Performance Status=0 (ECOG PS=0) (I and J), OS for Eastern Cooperative Oncology Group Performance Status=1 (ECOG PS=1) (K and L).

Supplementary Figure 7. Comparisons of the fit of consistency and inconsistency models using deviance information criteria (DIC).

DIC		Overall				6-Months	1-Year	HER-2+		HER-2-		Previous regimens(2)	Previous regimens(3)	Previous gastrectomy(Yes)	Previous gastrectomy(No)
	Model	PFS	OS	DCR	⩾3AEs	PFS rate	OS rate	OS	PFS	OS	PFS	OS	OS	OS	OS
Consisitency	Fixed	16.80	18.70	18.17	12.09	16.19	24.18	4.00	4.00	4.00	4.00	9.98	6.00	6.00	6.01
	Random	12.26	16.29	19.25	12.14	16.23	24.16	4.00	4.00	4.00	3.99	9.99	6.00	6.00	5.99
Inconsisitency	Fixed	16.79	18.71	17.95	12.03	16.07	24.02	4.00	3.99	3.99	3.99	9.99	5.99	6.01	5.98
DIC		ECOG PS=0	ECOG PS=1	Intestinal type	Diffuse type	Gastric	GEJ	Age ⩾65	Age<65	Male	Female	Metastasis site(1)	Metastasis sites(2)	Measurable lesion(No)	Measurable lesion(Yes)
	Model	OS	OS	OS	OS	OS	OS	OS	OS	OS	OS	OS	OS	OS	OS
Consisitency	Fixed	12.01	11.98	6.01	6.01	6.00	6.00	12.01	11.99	10.00	9.99	7.99	7.99	5.99	5.99
	Random	12.01	12.02	5.97	6.01	6.00	5.99	12.01	11.98	9.99	9.98	7.99	8.01	5.98	5.99
Inconsisitency	Fixed	12.00	12.00	5.99	5.98	6.01	5.99	12.02	11.97	9.99	9.98	8.00	7.99	5.99	6.01

The DIC is a Bayesian model evaluation criterion that measures model fit adjusted with complexity of the model; smaller DIC values correspond to more preferable models. Abbreviations: GEJ, Gastroesophageal junction.
